# Dimensions and Clusters of Aesthetic Emotions: A Semantic Profile Analysis

**DOI:** 10.3389/fpsyg.2021.667173

**Published:** 2021-05-28

**Authors:** Ursula Beermann, Georg Hosoya, Ines Schindler, Klaus R. Scherer, Michael Eid, Valentin Wagner, Winfried Menninghaus

**Affiliations:** ^1^Department of Psychology, UMIT–Private University for Health Sciences, Medical Informatics and Technology, Hall in Tirol, Austria; ^2^Department of Education and Psychology, Division of Methods and Evaluation, Freie Universität Berlin, Berlin, Germany; ^3^Department of Language and Literature, Max Planck Institute for Empirical Aesthetics, Frankfurt am Main, Germany; ^4^Department of Psychology, University of Geneva, Geneva, Switzerland; ^5^Department of Psychology, Ludwig-Maximilians-University Munich, Munich, Germany; ^6^Humanities and Social Sciences, Helmut Schmidt University/University of the Federal Armed Forces Hamburg, Hamburg, Germany

**Keywords:** aesthetic emotions, dimensional structure, emotion word clusters, affective meaning, semantic profiles

## Abstract

Aesthetic emotions are elicited by different sensory impressions generated by music, visual arts, literature, theater, film, or nature scenes. Recently, the AESTHEMOS scale has been developed to facilitate the empirical assessment of such emotions. In this article we report a semantic profile analysis of aesthetic emotion terms that had been used for the development of this scale, using the GRID approach. This method consists of obtaining ratings of emotion terms on a set of meaning facets (features) which represent five components of the emotion process (appraisal, bodily reactions, action tendencies, expression, and feelings). The aims here were (a) to determine the dimensionality of the GRID features when applied to aesthetic emotions and compare it to published results for emotion terms in general, and (b) to examine the internal organization of the domain of aesthetic emotion terms in order to identify salient clusters of these items based on the similarity of their feature profiles on the GRID. Exploratory Principal Component Analyses suggest a four-dimensional structure of the semantic space consisting of valence, power, arousal, and novelty, converging with earlier GRID studies on large sets of standard emotion terms. Using cluster analyses, 15 clusters of aesthetic emotion terms with similar GRID feature profiles were identified, revealing the internal organization of the aesthetic emotion terms domain and meaningful subgroups of aesthetic emotions. While replication for further languages is required, these findings provide a solid basis for further research and methodological development in the realm of aesthetic emotions.

## Introduction

When asked to describe an aesthetic experience (Leder et al., 2004) of a piece of music, an exhibition at a museum, a poem or novel, a theater play or movie, or a walk through nature, people often refer to emotions, for instance, by characterizing their experience as delightful, moving, interesting, funny, repulsive, or boring. Because of their relevance to aesthetic experience and evaluation, emotions such as these have been labeled “aesthetic emotions” (Frijda, 1989; Keltner and Haidt, 2003; Silvia, 2005, 2009; Scherer and Coutinho, 2013; Perlovsky, 2014; Menninghaus et al., 2019). But what exactly are people conveying when they use words that designate aesthetic emotions? Do emotion words take on a special semantic meaning in the context of aesthetics? Which aesthetic emotion words refer to similar emotional experiences, and what are the dimensions underlying such similarities?

We employed a psycholinguistic tool—the GRID instrument (Fontaine et al., 2013)—to shed more light on the semantic meaning of aesthetic emotion terms. According to the Component Process Model of Emotion (CPM, Scherer, 1984, 2005, 2009, 2013a), an emotion episode consists of dynamic changes in five components: subjective feelings, cognitive appraisals, bodily reactions, (facial, vocal, and postural) expressions, and action tendencies. The GRID instrument allows studying the lay concept behind an emotion term by asking which emotion component features respondents associate with this specific term. We studied a broad range of aesthetic emotion terms and determined their semantic profiles on features of the five emotion components. These profiles were employed to address two central research questions concerning the underlying dimensionality and internal organization of aesthetic emotion concepts: (1) What kind of dimensional structure best describes the semantic meaning of *aesthetic* emotion terms, as compared to what has been found in studies on other types of emotion terms in different contexts? (2) How is the domain of aesthetic emotions structured based on similarities in pertinent feelings, appraisals, bodily reactions, expressions, and action tendencies associated with each emotion term?

### Aesthetic Emotions

Many researchers share the assumption that emotions are centrally involved in aesthetic experiences. Under this assumption, it is of interest to study what is special about aesthetic emotions (Frijda, 1989; Keltner and Haidt, 2003; Scherer, 2004; Silvia, 2005, 2009; Scherer and Coutinho, 2013). However, the definition of the construct “aesthetic emotion” has remained controversial. In an extensive review, Menninghaus et al. (2019) proposed a definition of aesthetic emotions as intuitive evaluations of subjectively perceived aesthetic virtues and vices (see also Fingerhut and Prinz, 2020, Menninghaus et al., 2020). Specifically, four mandatory features define aesthetic emotions: they (1) include an aesthetic evaluation or appreciation of the respective events or objects, (2) are predictive of a certain type of aesthetic appeal, (3) are associated with a subjective feeling of pleasure or displeasure, and (4) predict liking or disliking of the event or object in question (Menninghaus et al., 2019, pp. 171–172).

Different measurement instruments have been implemented to assess aesthetic emotions (for an overview see Schindler et al., 2017). These include emotions elicited by music (e.g., Zentner et al., 2008; Peltola and Eerola, 2016; Schubert et al., 2016; Coutinho and Scherer, 2017; Crickmore, 2017), visual art (e.g., Silvia and Nusbaum, 2011; Augustin et al., 2012) and films (e.g., Renaud and Unz, 2006; Bartsch, 2012). However, emotion terms such as being moved, fascination, awe, and wonder—considered as typical aesthetic emotions (Keltner and Haidt, 2003; Scherer, 2004; Frijda, 2007; Scherer and Coutinho, 2013; Fingerhut and Prinz, 2020)—are not only reserved for questionnaires assessing aesthetic emotions, but are likewise used to characterize emotional experiences that do not involve aesthetic evaluations. Thus, we can conceive of aesthetic emotion terms as “conceptual blends” (Menninghaus et al., 2020): an available lexicalized emotion term is bestowed with a context-driven additional meaning. In the case of aesthetic emotions, emotion terms are used with the understanding that they communicate an additional aesthetic evaluation on top of feelings, appraisals, bodily reactions, expressions, and behaviors associated with this emotion.

In their study on word usage in visual aesthetics, Augustin et al. (2012) have deplored the lack of precision in terminology regarding the description and measurement of aesthetic impressions. They highlighted the need to identify the semantic interrelations of aesthetic emotion words and to find out about similarities and dissimilarities as well as possible nodes in terms of a semantic network, to advance empirical measurement. In the current study, we chose a semantic approach to investigate the meaning of emotion terms within an aesthetic context. We employed a set of 75 aesthetic emotion terms that has been investigated in two prior studies. Schindler et al. (2017) have developed the Aesthetic Emotions Scale (AESTHEMOS) as a domain-general instrument for assessing aesthetic emotions based on this set of 75 emotion terms (finally retaining 42 items). The emotion items were tested in a field study, for which participants were recruited after visiting an event of aesthetic interest (e.g., concert, film screening, theater performance, reading, or museum exhibition). The same pool of 75 emotion terms was employed by Hosoya et al. (2017) to investigate the conceptual domain of aesthetic emotions: In this study, participants sorted the emotion terms according to perceived similarities between them into as many piles as they wanted.

The results of these studies have provided first information on the internal structure of this set of aesthetic emotion terms based on self-reported experience and perceived semantic similarity. For this reason, we considered the same set of terms as ideally suited for the present study employing a semantic approach based on identifying the facets of meaning of words.

### Emotional Space and the GRID Paradigm

The question of a dimensional representation of the emotion domain has been controversially discussed for decades. Some researchers found evidence for a two-dimensional structure, typically consisting of valence and arousal (e.g., Russell, 1980; Watson and Tellegen, 1985; Feldman, 1995); others proposed a three-dimensional structure (Osgood et al., 1957; Shaver et al., 1987), namely valence, arousal, and power (or potency). In the domain of aesthetic emotions generated by music, Schubert and his collaborators (Schubert et al., 2016, 2020) have proposed a two dimensional model of the affect space, consisting of a valence dimension and an external/internal locus dimension (external—the expression of a particular emotion by a work of art as perceived by the observer and aesthetic judgments, internal—a particular emotional feeling experienced by the same observer in response to the work of art).

More recently, evidence was found for a four-dimensional structure, which, in addition to valence, arousal, and power, includes novelty (Fontaine et al., 2007, 2013, 2021; Gillioz et al., 2016; Gentsch et al., 2018). These studies used the GRID paradigm (Fontaine et al., 2013) to analyze semantic feature profiles of emotion terms. Furthermore, novelty was demonstrated as a fourth dimension by Fontaine and Veirman (2013), who employed a pairwise similarity rating task with a set of 85 emotion terms.

The GRID paradigm is based on Scherer's (2005, p. 707–712) suggestion to use a design feature analysis to explore the semantic structure of emotion terms by mapping the folk concepts of emotion as expressed in emotion words into the theoretical framework established by emotion psychology. This framework consists of the CPM (Scherer, 1984, 2005, 2009, 2013a), which proposes that an emotion process consists of dynamic unfolding of changes in the five emotion components listed above, in a recursive process driven by appraisal checks. These include: (1) relevance checks in respect to novelty, intrinsic pleasantness, and goals or needs, (2) implication checks concerning causal attribution, outcome probability, and discrepancy from expectations, (3) ways of coping with the respective event, and (4) normative significance checks like compatibility with internal and external standards (Scherer, 2009, p. 1313; Scherer, 2013b, p. 151). The feeling component monitors and regulates the component process, and it allows a person to communicate their emotional experience to other people. For each of these components, a number of specific design features can be defined, which serve to define the semantic profiles of the emotion words used in a language. As an example, in a lay person's understanding of the emotion term “happy,” this person has typical characteristics, or features, for this emotion in mind: for example, in that person's understanding, a happy person typically feels good (which is a feature of the subjective feeling component), smiles (a feature of the expression component) and might want to sing and dance (a feature of the behavior tendency component). This results in a two-dimensional grid table with the component features in rows and the emotion words in columns (which gave the “GRID” its name).

For research use, a GRID instrument has been designed that allows one to determine the pertinence of different semantic features for the meaning of a particular emotion word in a certain language by obtaining ratings on a 9-point scale by speakers of the respective language (see Table 1). The data obtained in this fashion serve to investigate the meaning and conceptualization of a specific emotion word, such as anger or surprise, and to compare these across languages and cultures. The GRID instrument has been used in a number of cross-cultural and cross-language studies (34 samples, over 28 languages in 31 countries), data were obtained with 142 (FullGRID instrument) or 68 semantic features (CoreGRID instrument; Scherer et al., 2013) and a set of 24 emotion words that had been selected based on frequent reference to them in emotion research and on their usage in everyday communication (Fontaine et al., 2007, 2013; for a recent replication with a larger set of emotion terms, see Gillioz et al., 2016; Fontaine et al., 2021), and—as an additional specific emotion domain using a different set of emotion words—in achievement emotions (Achievement Emotion GRID, Gentsch et al., 2018; achievement emotions refer to emotions occurring in academic and achievement contexts, Pekrun, 2006).

**Table 1:** An example of a component-based semantic grid (adapted from Fontaine et al., 2013, p. 27).

**Emotions**	**Anger**	**Fear**	**Shame**	**Guilt**
**Features**				
The event was unpredictable	2	7	6	2
The event was caused by somebody else's behavior	8	2	5	2
The event had negative, undesirable consequences for the person	7	4	5	1
Rapid heart rate	7	9	4	2
Feeling warm	6	2	8	2
Smiled	1	1	4	1
Wanted to do damage, hit, or say something that hurts	7	3	1	1
Wanted to disappear or hide from others	2	4	6	8

*The numbers in the cells denote how likely the respective feature is attributed to an emotional experience as labeled with the emotion words in the top row of the table (on a scale from 1 to 9; Fontaine et al., 2013, p. 27)*.

Apart from interesting specificities concerning language and regional differences, a stable four-dimensional structure consisting of valence, power, arousal, and novelty emerged across all languages and cultures (Fontaine et al., 2007, 2013, 2021; Gillioz et al., 2016), and within the specific domain of achievement emotions (Gentsch et al., 2018), empirically establishing that these four dimensions are necessary to meaningfully differentiate between the wide range of emotion terms existing in different languages. So far, there is no evidence that the four-dimensional structure holds also true for the domain of *aesthetic* emotions. One might expect differences in the semantic structure of aesthetic emotions because they differ from emotions in other—everyday—domains concerning their appraisals of goal relevance and coping potential (Scherer and Zentner, 2008; Scherer and Coutinho, 2013; see also Lajante et al., 2020). Therefore, the current study investigates the number and nature of dimensions of the semantic space in the realm of aesthetic emotions.

### Aims of the Study and Research Questions

The current study used the GRID paradigm to study the semantic meaning of aesthetic emotion terms. Our first research question concerned the dimensionality of the GRID features when applied to aesthetic emotion terms as compared to the one obtained in prior GRID studies (Scherer et al., 2013; Gillioz et al., 2016; Gentsch et al., 2018; Fontaine et al., 2021). We investigated whether the same four dimensions that were obtained when investigating emotion words used in everyday language or achievement emotions—valence, power, arousal, and novelty—can also be identified in the field of aesthetic emotions, or whether the dimensionality in the realm of aesthetic emotions differs from other domains of emotion.

Our second question concerned similarities between aesthetic emotion terms. Our goal was to get a better understanding of the internal organization that laypeople have of aesthetic emotion concepts in terms of their semantic profiles as represented by the GRID features. These features allow us to examine which of the five emotion components are typically associated with an aesthetic emotion, going beyond just studying associations of the items amongst themselves and thus adding another dimension of meaning that provides insight on how emotion items are understood by laypeople in terms of the components of the CPM (Scherer, 1984, 2005, 2009, 2013a). Clustering the items based on their semantic profiles helps to further elucidate the internal organization of the item set.

## Materials and Methods

### Ethics Statement

The study was part of a larger research project (see Funding information) for which the Ethics Committee of the University of Geneva, the host of the project, approved the research procedures. Participation was voluntary and anonymous. The participants gave informed consent by clicking on the “next” button after receiving information about the study. They were free to withdraw their consent to participate at any time.

### Participants

In total, 157 students of the Freie Universität Berlin participated (38 males, 119 females, aged between 17 and 55 years, *M* = 24.25, *SD* = 5.28). Of the 157 participants, 140 grew up speaking German. Seventy-five participants of the sample were active in an artistic domain (such as singing, playing an instrument, writing, creating visual art, dancing, or acting).

### Material

#### Emotion Terms

The original cross-cultural GRID study used 24 emotion words (only nouns, such as “disgust” or “anger”; see Fontaine et al., 2013). In the follow-up studies, 80 emotion words were used (only nouns; Gillioz et al., 2016; Fontaine et al., 2021). Given the special context of widely varying aesthetic emotion experiences, we did not use the familiar nouns for major emotions from prior GRID studies for developing the Aesthetic Emotion GRID, but rather the same pool of 75 emotion terms that were already used in two prior studies (Hosoya et al., 2017; Schindler et al., 2017; see section “Aesthetic Emotions” of the Introduction section). These emotion terms consist of words or short phrases describing the type of emotional experience. Arguably, they provide a comprehensive pool of idiomatic expressions for aesthetic emotion experiences. Furthermore, we added an aesthetic context to the rating study by instructing the participants to imagine someone who had just had an aesthetic experience. Aesthetic experiences were illustrated with examples (e.g., perception of objects such as paintings, literature, musical, theatrical, or movie performances, or appearances such as a sunset), suggesting to participants that these may elicit emotions which are described with certain words of their language. These words, in turn, provide information on the response of a person to the aesthetic experience. Participants were informed that the goal of the study is to help characterize the meaning of these emotion words in terms of certain characteristics such as facial expressions, bodily changes, or behavioral tendencies connected with these words. The emotion terms were presented in German (for German and English versions see Supplementary Table 1).

#### GRID Features

Whereas, the original GRID study used 142 semantic features, the follow-up studies used a reduced set of 68 features that had been found to be most discriminating (the CoreGRID instrument, Scherer et al., 2013). We also used the CoreGRID features grouped into five emotion components: (1) *subjective feelings* (10 features; e.g., “good”), (2) *bodily reactions* (11 features; e.g., “rapid heart rate”), (3) *facial, vocal, and postural expression* (12 features; e.g., “spoke more loudly”), (4) *behavior tendencies* (14 features; e.g., “wanted to sing and dance”), and (5) *event evaluations/appraisals* (cognitive evaluations of the situation or event; 21 features; e.g., “the event was uncontrollable”; for German and English versions of the features see Supplementary Table 2). While 66 of the features are expected to yield distinct profiles for individual emotions, two of the features represent general qualifiers of emotions: the feature “[felt] the emotion very intensely,” and “[felt] the emotion for a long time.” These two features were excluded from all analyses, as they are not expected to contribute substantially to the semantic differentiation, resulting in 66 features to be analyzed.

### Procedure

The Aesthetic Emotion GRID was presented in a software interface developed and hosted at the Swiss Center for Affective Sciences (CISA) at the University of Geneva. The data were collected in group sessions that took place in a seminar room of the Freie Universität Berlin. The participants completed the survey on computers with the questionnaire running in a web browser. First, the participants answered a series of background questions. Subsequently, they rated the degree to which each of the features applies to each emotion term. The scale ranged from *extremely unlikely* (1) to *extremely likely* (9).

We took great care to make the rating procedure as untiring for the participants as possible. To make the task feasible, the set of emotion terms was divided into 10 groups, or lists, each comprising seven or eight emotion terms to be rated by the participants. The terms in each group represented different emotion categories (e.g., positive/negative valence, nostalgia, activation, impact on a person, sadness, humor). Similar emotion terms, such as “worried me” and “made me anxious,” were distributed into different emotion groups (for the assignment of emotion terms to groups see Supplementary Table 1). Each participant rated only the emotion terms of one group on all CoreGRID features throughout the study (each term was rated by 15–17 participants). The assignment of the participants to 10 groups was permuted (e.g., participant 1 was assigned to group 1, participant 2 to group 2 etc.) to assure appropriate randomization. Native vs. non-native German speakers were equally distributed across the groups, χ^2^(9, 157) = 12.83, *p* = 0.17, n.s. Likewise, participants with and without artistic background were equally distributed across the groups, χ^2^(9, 157) = 7.19, *p* = 0.62, n.s.

The features to be rated were grouped in categories according to the Component Process Model of Emotion (CPM, Scherer, 1984, 2005, 2009, 2013a). That is, participants rated the same set of 7–8 emotion terms first on features of the category “subjective feelings,” then “bodily reactions,” then “facial, vocal and postural expression,” then “behavior tendencies,” and then “event evaluation.” This sequence of categories was the same for all participants because—based on experience from previous studies—this sequence of categories is the easiest for participants to rate. Within each of these categories, participants received the same instruction on the top of the page. For instance, for subjective feelings the instruction was: “If a person uses the following emotion term (in the left-hand column) to describe an emotion during or after having (had) an aesthetic experience, how likely is it that this person …” (followed by a feature; see Supplementary Figure 1 as an illustration of the task).

Below the instruction, participants were presented with a table containing the list of 7–8 emotion terms in the left column (e.g., “find it sublime”) and a rating scale from 1 (extremely unlikely) to 9 (extremely likely) in the top row (See Supplementary Figure 1). In order to enhance readability, within each category, for each participant, the sequence of emotion terms stayed the same. However, the sequence of emotion terms was randomized across the participants of the same group. Also, for a given participant, the sequence of emotion terms changed from category to category. Above this table, one by one, a feature was presented (e.g., “good,” “bad,” “strong”). To give an example, participants were asked: If a person uses the emotion term “find it sublime” to describe an emotion during or after having (had) an aesthetic experience, how likely is it that this person “closed their eyes”?. Each new feature was presented on a new page, so that the only word that changed within one category from page to page was the feature, in order to minimize cognitive effort when performing the task. Within each category, the sequence of the presented features was randomized across participants.

The whole procedure took ~45–60 min. Participants received a monetary compensation of 10€.

### Data Preparation

We followed the procedure established by Fontaine et al. (2013), which takes into consideration that the capability of laypeople of identifying the meaning of emotion terms may differ. First, for each emotion term, inter-rater consistency (Cronbach's α) and corrected item total correlations for each rater (*citc*; i.e., the correlation of a participant's profile with the average profile of the other participants) were established across all raters of each list. Participants who had a *citc* of <0.20 were removed from all further analyses for this particular emotion term rating (Fontaine et al., 2013, p. 102). The procedure was repeated until all remaining participants had a *citc* of at least 0.20. Per emotion term, the ratings of between 0 and 6 participants were removed (*M* = 0.93, *SD* = 1.24; for the number of total and remaining participants for each emotion term see Supplementary Table 1). Ratings by non-native speakers were more likely to be excluded than by native speakers [χ^2^ (1, 157) = 7.11, *p* = 0.008], and ratings by participants without artistic background were more likely to be excluded than ratings by participants with artistic background [χ^2^ (1, 157) = 4.13, *p* = 0.04]. The inter-rater consistency (Cronbach's α) after this procedure ranged from α = 0.72 (“Filled me with longing”) to α = 0.96 (“Calmed me” and “I found it pleasant”) with an average Cronbach's α of 0.90 (for Cronbach's α for each emotion term before and after the removal of participants see Supplementary Table 1).

Subsequently, the data were centered as follows: First, the data were aggregated across the participants such that each line of the resulting dataset represented one emotion term and each column represented a GRID feature, rendering the emotion terms the “cases” of our data set. Then, a mean score was calculated across the 66 features for each emotion term. This mean score was subtracted from each feature score of the respective emotion term (see Fontaine et al., 2013, p. 93). The resulting data set and the analyses scripts are available online (https://osf.io/8f6em/).

## Results

### Dimensional Structure of the GRID Features

In order to investigate the dimensional structure of the GRID features in the domain of aesthetic emotions, we used an exploratory approach to allow to determine potential specificities of the aesthetic emotion space. In accordance with previous GRID studies (Fontaine et al., 2007, 2013, 2021; Gillioz et al., 2016; Gentsch et al., 2018), and because the dataset has a relatively low number of cases (75 emotion terms) as compared with variables (66 GRID features), we therefore conducted a Principal Component Analysis (PCA) based on Pearson correlations. The PCA was computed with SPSS (Version 24) across the 66 centered feature scores of the Aesthetic Emotions GRID.

The Kaiser–Meyer-Olkin measure of sampling adequacy (KMO) was mediocre with KMO = 0.62 (Kaiser, 1974). Bartlett's test of sphericity was χ^2^ (2,145) = 9,625.54, *p* < 0.001 (for the uncentered data), indicating that the correlations between items were sufficiently large for calculating a PCA. As there are no clear-cut criteria for choosing the appropriate cut-off point for the number of dimensions to be extracted, we determined the range of possible solutions given accepted cut-off criteria. The Eigenvalue > 1 criterion indicated five dimensions to be extracted (the Eigenvalues being 32.50, 16.80, 4.05, 2.46, and 1.41; note that in order not to confuse the term “component” with the “emotion components” according to Scherer's Component Process Model of Emotion, we used the term “dimension” instead of “component” to describe the results of the PCA. Likewise, we used “feature scores” instead of “component scores” and “feature loadings” instead of “component loadings”). The “knee” in the scree plot supported a four-dimensional solution (See Supplementary Figure 2). Horn's parallel analysis (Horn, 1965) yielded three observed eigenvalues that exceeded the 95th percentile eigenvalues, barely missing the 95th percentile for the fourth eigenvalue. Thus, solutions from three to five dimensions are conceivable.

The three-dimensional solution explains 80.84% of variance and the dimensions can be interpreted as valence, arousal, and power (see Osgood et al., 1957, Shaver et al., 1987). As in the earlier work (Fontaine et al., 2007, 2013, 2021; Gillioz et al., 2016), the three-dimensional solution seems rather limited with respect to the differentiation it affords. The four-dimensional solution explains 84.56% of total variance, and yields dimensions that can be interpreted as valence, power, arousal, and novelty, corresponding largely to the earlier findings on general emotion terms. The five-dimensional solution is defensible but affords only a very minor gain in additional variance explained (86.69% of total variance explained, which is only 2.13% more than the four-dimensional solution). Furthermore, it basically splits the power dimension into two power subdimensions: the five dimensions can be interpreted as valence, arousal, power motivation (such as wanting—or not wanting—to tackle a situation or to overcome an obstacle, but also being strong or weak), power potential (such as having—or lacking—the power and control over the consequences of an event), and novelty. This subdivision of the power dimension is of little relevance for the aesthetic emotion domain as the issue of coping potential (high or low power to control or cope with the consequences of an aesthetic experience) is of relatively little importance in this domain. The novelty dimension is part of both the four- and five-dimensional solutions. The role of novelty in the realm of aesthetic emotions has been emphasized by several researchers (e.g., Fayn et al., 2015; Menninghaus et al., 2019). Further considering the notion that the Eigenvalue > 1 criterion typically leads to an extraction of too many dimensions (e.g., Field, 2009), we consequently settled on the four-dimensional solution for our further analyses, especially as it reflects the earlier findings in dedicated semantic analyses of emotion terms (however, the loading tables for the rotated three- and the five-dimensional solutions can be viewed in Supplementary Tables 3, 4, respectively).

As the features are expected to differentially load on several dimensions (see chapter 7 in Fontaine et al., 2013), we chose a rotation well-suited for complex data structures, in this case Equamax (which combines Varimax and Quartimax, optimizing both column and row variance; Schmitt and Sass, 2011, p. 110), which is arguably the best option to obtain an unbiased result. The resulting feature loadings as well as the communalities are shown in Table 2. The four dimensions can be readily interpreted as valence, power, arousal, and novelty, converging with the earlier GRID findings both with respect to the nature of the dimensions and the sequence. The feature loadings (presented in Table 2) indicate that the two dimensions valence and power are inverted: for *valence*, positive loadings represent negative valence; for *power*, positive loadings stand for low power, and vice versa.

**Table 2:** Rotated component matrix of the GRID features resulting from a principal component analyses using orthogonal equamax rotation, extracting 4 dimensions.

**Nr**.	**Feature**	**Comp**.	**Comm**.	**Valence**	**Power**	**Arousal**	**Novelty**
34	Wanted the ongoing situation to last or be repeated	Be	0.978	**−0.861**	−0.446	0.076	−0.181
63	The event was inconsistent with the person's own standards and ideals	Ev	0.935	**0.849**	0.426	0.088	0.158
35	Wanted to stop what he/she was doing	Be	0.897	**0.845**	0.366	−0.202	0.085
36	Wanted to undo what was happening	Be	0.944	**0.836**	0.482	−0.076	0.091
42	Wanted to do damage, hit, or say something that hurts	Be	0.856	**0.831**	0.312	0.211	0.154
51	The event was pleasant for the person	Ev	0.977	**−0.827**	−0.505	−0.004	−0.196
22	Smiled	Ex	0.951	**−0.824**	−0.488	0.013	−0.182
8	Bad	F	0.946	**0.819**	0.503	−0.122	0.087
3	Good	F	0.972	**−0.818**	−0.522	−0.011	−0.174
43	Wanted to oppose someone or something	Be	0.880	**0.813**	0.144	0.440	0.068
58	The event had negative, undesirable consequences for the person	Ev	0.950	**0.811**	0.521	0.070	0.123
19	Feeling warm	Bo	0.906	**−0.804**	−0.429	0.040	−0.271
47	Wanted to sing and dance	Be	0.920	**−0.801**	−0.467	0.182	−0.166
25	Frowned	Ex	0.796	**0.779**	0.138	−0.040	0.411
46	Wanted to run away in any direction	Be	0.923	**0.769**	0.550	0.141	0.096
64	The event involved the violation of socially accepted norms	Ev	0.840	**0.758**	0.393	0.197	0.270
13	Stomach disturbance	Bo	0.947	**0.756**	0.588	0.032	0.166
21	Feeling cold	Bo	0.898	**0.734**	0.478	−0.353	0.082
52	The event was important for and relevant to the person's goals or needs	Ev	0.839	**−0.730**	−0.387	0.296	−0.263
41	Wanted to disappear or hide from others	Be	0.921	**0.718**	0.612	−0.166	0.058
40	Lacked the motivation to pay attention to what was happening	Be	0.852	**0.671**	0.175	−0.609	−0.016
5	Restless	F	0.861	**0.652**	0.405	0.404	0.332
62	The person could live with the consequences of the event	Ev	0.809	**−0.648**	−0.503	−0.231	−0.287
53	The event was important for and relevant to the goals or needs of somebody else	Ev	0.619	**−0.641**	−0.431	0.144	−0.044
67	There was no urgency in the situation involving the event	Ev	0.876	**−0.600**	−0.382	−0.523	−0.311
10	Awake	F	0.805	**−0.534**	−0.514	0.505	0.042
37	Wanted to comply with someone else's wishes	Be	0.568	**−0.516**	−0.136	−0.509	−0.156
29	Spoke in a trembling voice	Ex	0.892	0.162	**0.887**	0.233	0.158
30	Spoke in a firm voice	Ex	0.878	−0.322	**−0.833**	0.228	−0.169
66	The person had a dominant role in the situation involving the event	Ev	0.855	−0.376	**−0.771**	0.164	−0.302
11	Feeling weak in the limbs	Bo	0.882	0.362	**0.745**	−0.442	0.037
31	Had speech disturbances	Ex	0.790	0.139	**0.740**	0.196	0.430
27	Had tears in the eyes	Ex	0.845	−0.378	**0.739**	0.152	−0.364
61	The person had control over the consequences of the event	Ev	0.918	−0.450	**−0.737**	−0.073	−0.408
65	The person was powerless in this situation involving the event	Ev	0.861	0.519	**0.736**	−0.031	0.220
60	The person had power over the consequences of the event	Ev	0.857	−0.456	**−0.704**	0.014	−0.391
12	Becoming pale	Bo	0.873	0.566	**0.703**	−0.101	0.220
6	Strong	F	0.908	−0.629	**−0.632**	0.293	−0.163
9	Weak	F	0.850	0.533	**0.608**	−0.442	−0.006
68	The event was uncontrollable	Ev	0.822	0.426	**0.591**	0.232	0.488
55	The event was caused by the person's own behavior	Ev	0.753	−0.305	**−0.590**	−0.037	−0.557
50	The event confirmed the expectations of the person	Ev	0.856	−0.488	**−0.575**	−0.240	−0.479
44	Wanted to tackle the situation	Be	0.785	−0.429	**−0.574**	0.513	−0.089
57	The event had consequences that were predictable	Ev	0.640	−0.162	**−0.485**	−0.446	−0.424
39	Wanted to do nothing	Be	0.871	0.054	0.171	**−0.892**	−0.209
4	Tired	F	0.901	0.315	−0.052	**−0.877**	−0.175
15	Rapid heart rate	Bo	0.910	−0.191	0.131	**0.875**	0.300
14	Slowed heart rate	Bo	0.944	−0.150	−0.192	**−0.843**	−0.417
33	Spoke more slowly	Ex	0.895	−0.117	0.119	**−0.829**	−0.423
18	Rapid breathing	Bo	0.856	−0.089	0.175	**0.826**	0.369
17	Slowed breathing	Bo	0.896	−0.210	−0.145	**−0.805**	−0.428
16	Tense muscles	Bo	0.831	0.205	0.117	**0.786**	0.397
32	Spoke more rapidly	Ex	0.855	−0.216	−0.259	**0.775**	0.376
38	Wanted someone else to take the initiative	Be	0.766	0.314	0.296	**−0.759**	0.055
20	Sweating	Bo	0.796	0.280	0.280	**0.725**	0.337
28	Spoke more loudly	Ex	0.802	−0.116	−0.421	**0.698**	0.353
45	Wanted to overcome an obstacle	Be	0.727	0.000	−0.505	**0.682**	−0.084
59	The event required an immediate response	Ev	0.760	0.440	−0.074	**0.682**	0.309
26	Closed the eyes	Ex	0.832	−0.216	0.166	**−0.659**	−0.568
7	Calm	F	0.912	−0.504	−0.371	**−0.579**	−0.430
24	Raised the eyebrows	Ex	0.807	0.196	−0.109	0.264	**0.829**
49	The event was unpredictable	Ev	0.803	−0.010	0.169	0.450	**0.756**
23	Dropped their jaw	Ex	0.678	−0.198	0.071	0.312	**0.732**
54	The event happened by chance	Ev	0.755	−0.398	−0.107	0.292	**0.707**
48	The event occurred suddenly	Ev	0.829	−0.217	0.055	0.533	**0.704**
56	The event was caused by somebody else's behavior	Ev	0.451	0.154	0.278	0.046	**0.590**

*Comp., Component according to the CPM; Comm., Communality; F, Subjective Feeling; Bo, Bodily Reactions; Ex, Expression; Be, Behavior Tendencies; Ev, Event Evaluation. Boldface indicates the highest loading of each feature*.

**Figure 1:**
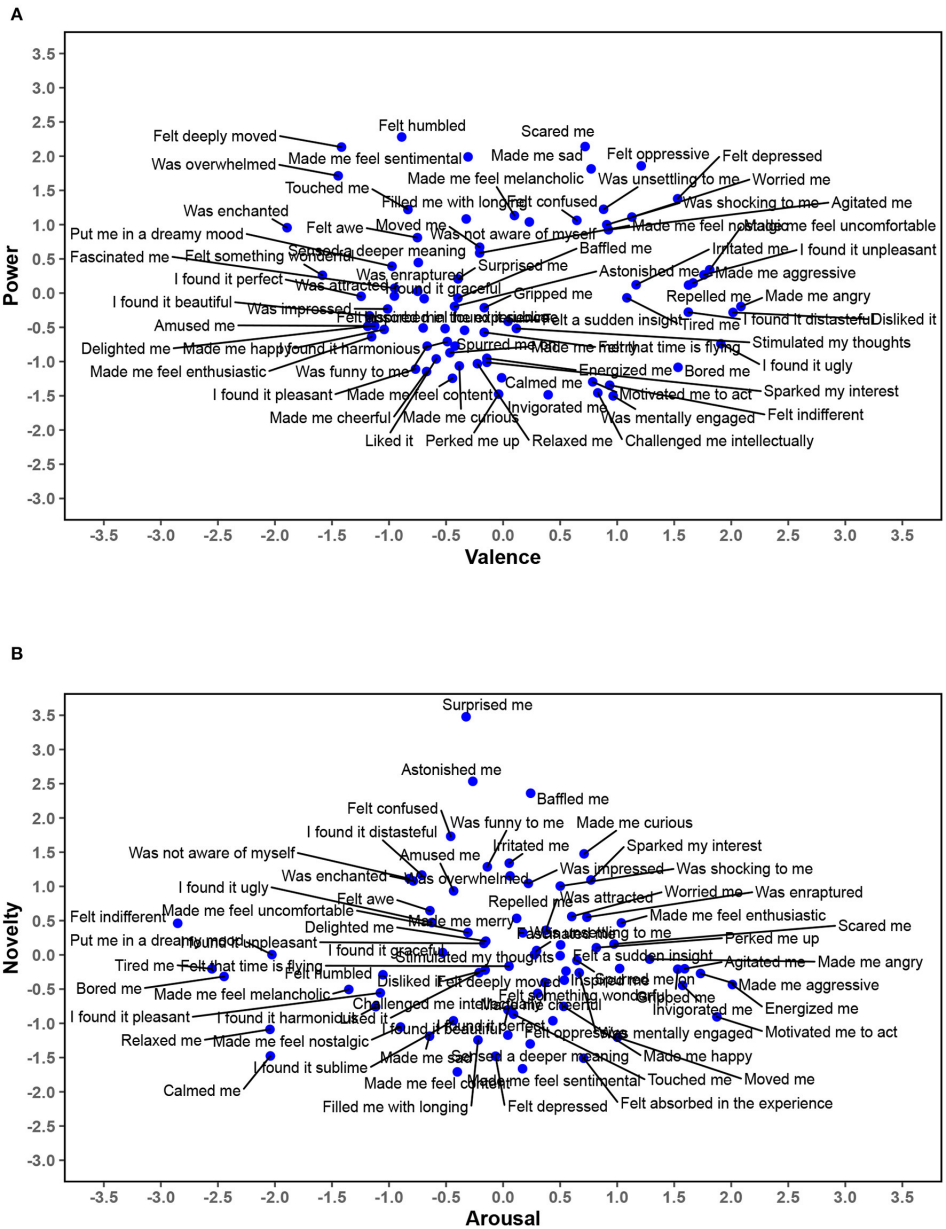
Feature scores of the 75 emotion terms represented by the four-dimensional structure. The two panels show scatterplots of the feature scores of Valence × Power **(A)** and Arousal × Novelty **(B)**. This figure has been generated using the R-packages *ggplot2* (Wickham, 2016) and svglite (Wickham et al., 2020) in RStudio (RStudio Team, 2019) and the open source vector graphics editor Inkscape (Bah, 2020).

In order to visualize where the emotion terms are situated on the four dimensions, we calculated feature scores (using the Anderson-Rubin method to conform with the orthogonal structure of the dimensions; DiStefano et al., 2009; note that the emotion terms—and not the participants—were of interest and represent the cases of the data set, see section Data Preparation of the Method section). The panels of Figure 1 show the scores on each feature of the emotion terms plotted against each other for *valence* × *power* and for *arousal* × *novelty*. The plots illustrating the dimensions *valence* x *arousal, power* × *arousal* and *arousal* × *novelty* can be found in Supplementary Figure 3.

In more detail, the two highest features for the *valence* dimension were “wanted the ongoing situation to last or be repeated” (positive valence) and “The event was inconsistent with the person's own standards and ideals” (negative valence). Emotion terms such as “felt something wonderful” and “amused me” were located on the high end of the *valence* dimension, and they were opposed by emotion terms referring to feelings of repulsion, anger, and uncomfortableness on its low end (Figure 1A).

For *power*, the highest features were “spoke in a trembling voice” and “spoke in a firm voice” (representing low and high power, respectively; Table 2). The power dimension was characterized for example by emotion terms referring to feeling humbled, deeply moved, or scared, on the low pole, and emotion terms expressing invigoration and motivation to act, as well as feeling indifferent and bored, on the high pole of power (Figure 1A).

The two highest features for *arousal* were “wanted to do nothing” and “tired” (both low arousal, see Table 2). As Figure 1B illustrate, emotion terms like energy, motivation to act, agitation, and aggression marked high arousal and were opposed by, for example, feeling indifferent and bored, feelings of relaxation, being calm, and being put in a dreamy mood.

In the dimension *novelty*, the two features with the highest loadings were “raised the eyebrows” and “the event was unpredictable” (all main loadings on novelty were positive). The most salient emotion terms on the high end of the novelty dimension were (not surprisingly) feelings of surprise and astonishment, along with confusion, funniness, and curiosity (Figure 1B). On the low end, novelty was represented by feelings of content, sentimentality, sadness, and being absorbed in the experience.

There were relatively high cross loadings for some of the features on other dimensions, such as the features “The event was pleasant for the person” or “good” of the valence dimension which also loaded on *power*, or “The event occurred suddenly” of the novelty dimension that also loaded on *arousal*. Nevertheless, both the features and the emotion terms representing the dimensions within the four-dimensional solution justified their overall interpretation as valence, power, arousal, and novelty.

### Aesthetic Emotion Clusters

To assess the similarities of the emotion terms in terms of ratings on the GRID features, explore their internal organization, and identify, as it were, “families” of aesthetic emotions, we conducted an exploratory *k*-means cluster analysis using the R programming environment for statistical computing (R Core Team, 2018). The input variables were the centered and aggregated features and the emotion terms represented the cases of the matrix. Euclidean distances were employed as is standard in k-means clustering. In a first step, the number of clusters was determined by examining the gap statistic (Tibshirani et al., 2001). The gap statistic calculates the difference between the *expected* log-pooled within-cluster sum of squares around the cluster means of data that were drawn from a reference distribution with no clustering structure, and those based on the *actual* data set. To calculate the gap statistic, we used the R-function clusGap() in the R-package “cluster” (Maechler et al., 2018). The principal component method was used to generate the reference data (Tibshirani et al., 2001) and 100 bootstrap replications were drawn from the reference distribution for each number of clusters *k*. Supplementary Figure 4 shows the gap statistic and the respective bootstrapped simulation errors for up to 50 clusters.

**Figure 2:**
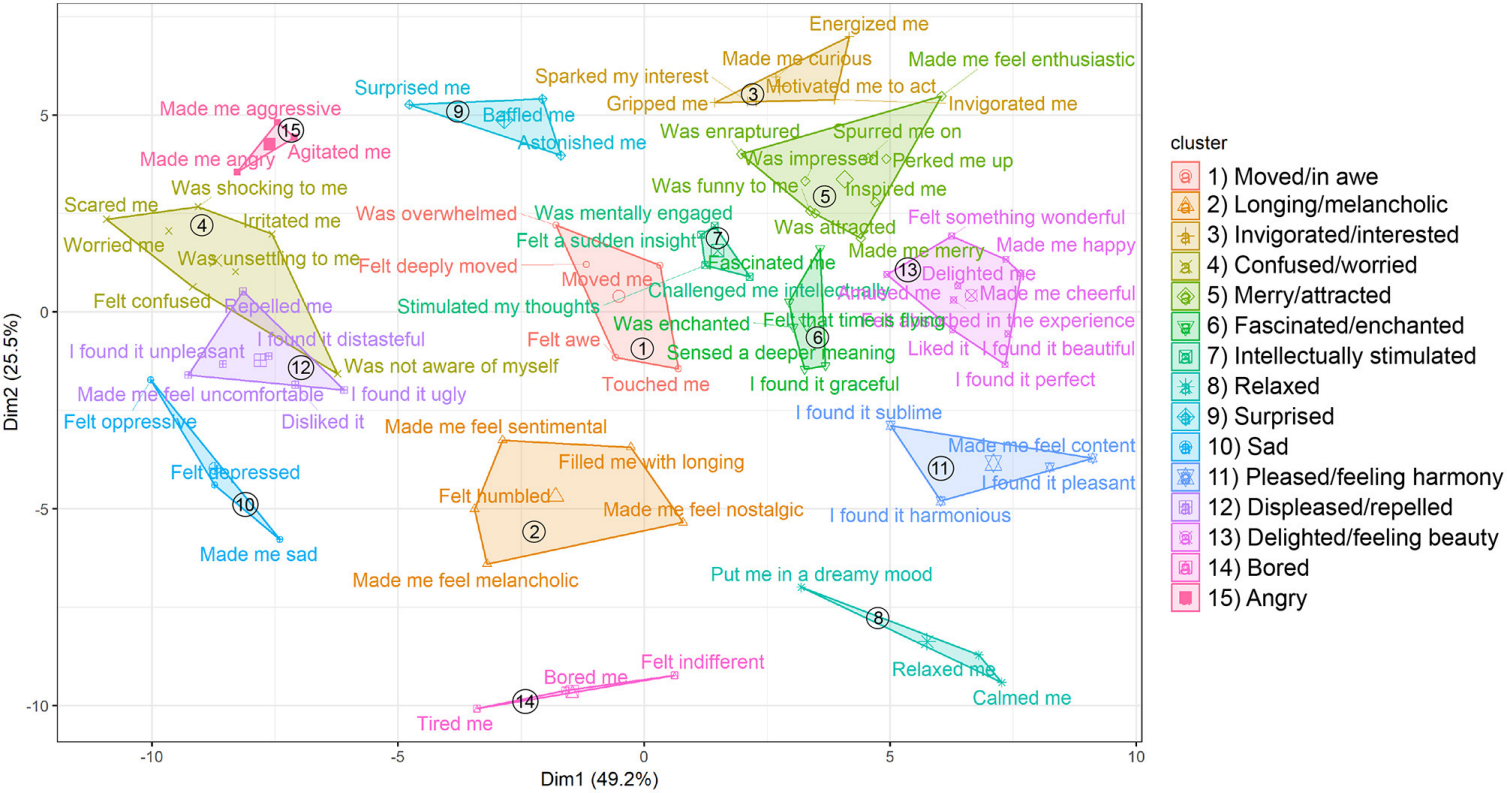
The 15 clusters derived from the k-Means clustering of the emotion terms. The graph was obtained using the R-package “factoextra” (Kassambara and Mundt, 2018).

In order to avoid an overfit with too many clusters (i.e., the number of clusters $\hat{k}$ with the maximum gap statistic, which would result in 49 clusters) or underfit (the minimum $\hat{k}$ accounting for the simulation error which was obtained via bootstrapping; see Tibshirani et al., 2001), which would result in a too parsimonious solution of 2 clusters, see Supplementary Figure 4), we chose the number of clusters in such a way that $\hat{k}$ is the smallest *k* located within one standard simulation error of the first local maximum. This method is implemented in the R-function maxSE() in the “cluster” package and can be used with the argument “firstSEmax.” Figure 2 shows the results of a *k*-means clustering for 15 clusters in which the items are projected onto a plane for interpretability (The graphs in Figure 2 and Supplementary Figure 4 were obtained using the R-package “factoextra”, Kassambara and Mundt, 2018). We want to emphasize that the solution is not meant to be a “proof” that the AESTHEMOS items are partitioned into 15 clusters in a population. The choice of the number of clusters is a complex procedure that involves a number of decisions. In the present case, theoretical considerations, the interpretability of the cluster solution, as well as the gap statistic were used. For this reason, the presented solution is an exploratory heuristic with the aim to gain a better understanding of the internal structure of the items in terms of GRID features.

The cluster center scores for each GRID feature are reported as part of the Supplementary Table 5. The cluster stability was determined with the Jaccard coefficient (Henning, 2007) using the bootstrapping method with 500 iterations. The function “clusterboot” of the R-package “fpc” (Henning, 2016) was used for these analyses. As a guideline for the interpretation of the coefficients, values below 0.60 are considered as not to be trusted, values between 0.60 and 0.75 indicate patterns in the data, values higher than 0.75 are regarded as stable and values higher than 0.85 as highly stable (Henning, 2016). As can be seen in Supplementary Table 6, the mean Jaccard similarity values range from 0.68 to 0.95, with the majority of clusters yielding coefficients higher than 0.75 and six clusters higher than 0.85, indicating a high stability of the clusters.

In Figure 2, the clusters are ordered along two dimensions, which can be best interpreted as valence (X-Axis, dimension 1) and arousal (Y-Axis, dimension 2). The suggested clusters are listed next to the plot, numbered and labeled with one or two emotion terms. The clusters shown on the left side of the plot−15 “angry,” 4 “confused/worried,” 12 “displeased/repelled,” and 10 “sad”—mark the negative pole of the dimension, while the clusters 5 “merry/attracted,” 13 “delighted/feeling beauty,” and 11 “pleased/feeling harmony” signify the positive pole. On dimension 2, the cluster 14 “bored” and 8 “relaxed” are located on the low end of arousal, whereas the clusters 3 “invigorated/interested” and 9 “surprised” are on the high end. However, as illustrated in particular by the clusters in the middle part of Figure 2, the dimensions valence and arousal are insufficient to account for all differences between the clusters. This applies specifically to clusters 1 “moved/in awe,” 2 “longing/melancholic,” and 7 “intellectually stimulated,” which include several emotions that have been seen as prototypical aesthetic emotions (see Schindler et al., 2017).

**Figure 3:**
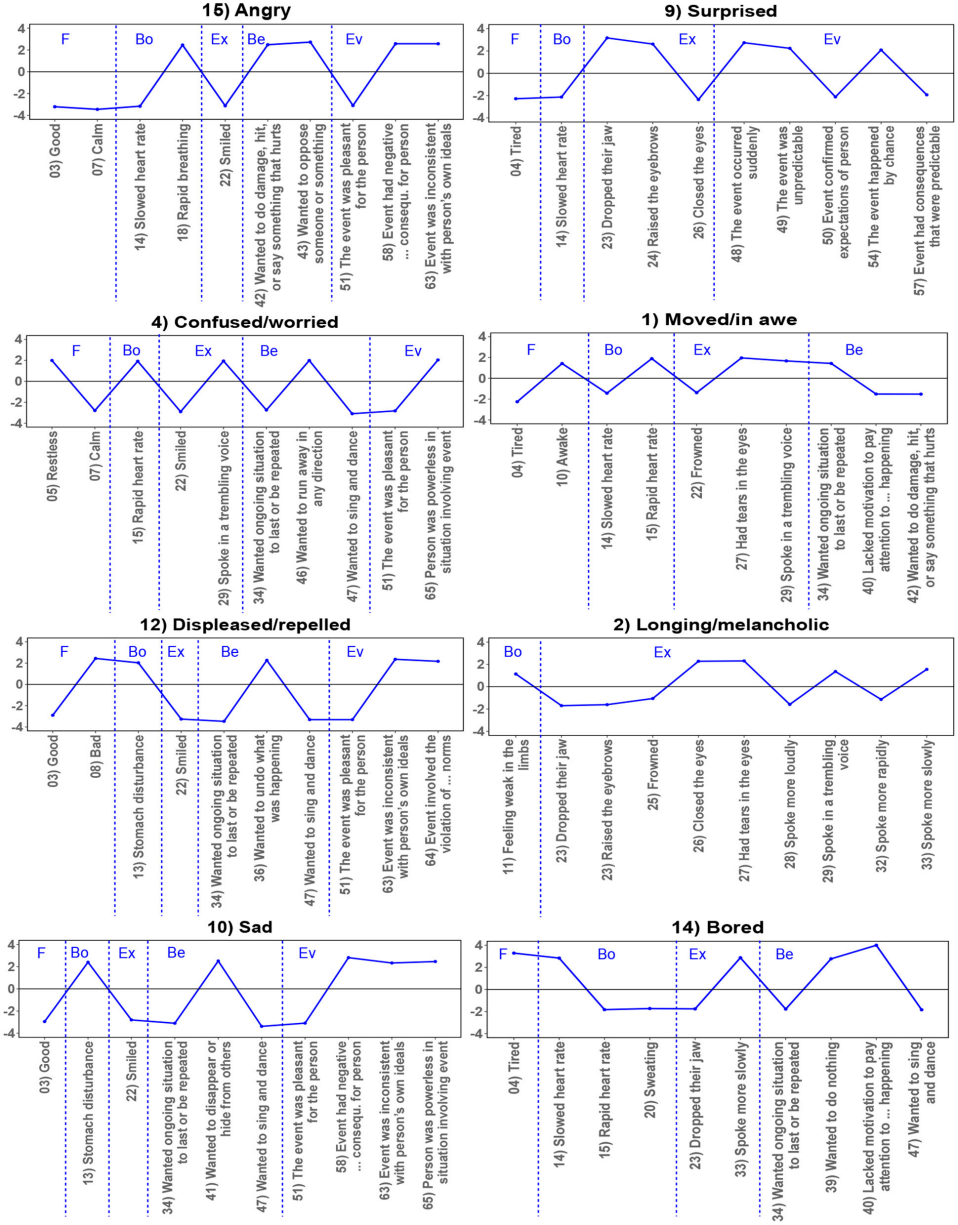
Profiles displaying the top and bottom 5 features for the clusters indicating (rather) negative valence, allocated approximately according to their position in the left half of Figure 2. Features are partly abbreviated and can be read in full in Supplementary Tables 1, 2. F, Subjective Feeling; Bo, Bodily Reactions; Ex, Expression; Be, Behavior Tendencies; Ev, Event Evaluation. The figure has been generated using the R-packages *ggplot2* (Wickham, 2016) and svglite (Wickham et al., 2020) in RStudio (RStudio Team, 2019) and the open source vector graphics editor Inkscape (Bah, 2020).

**Figure 4:**
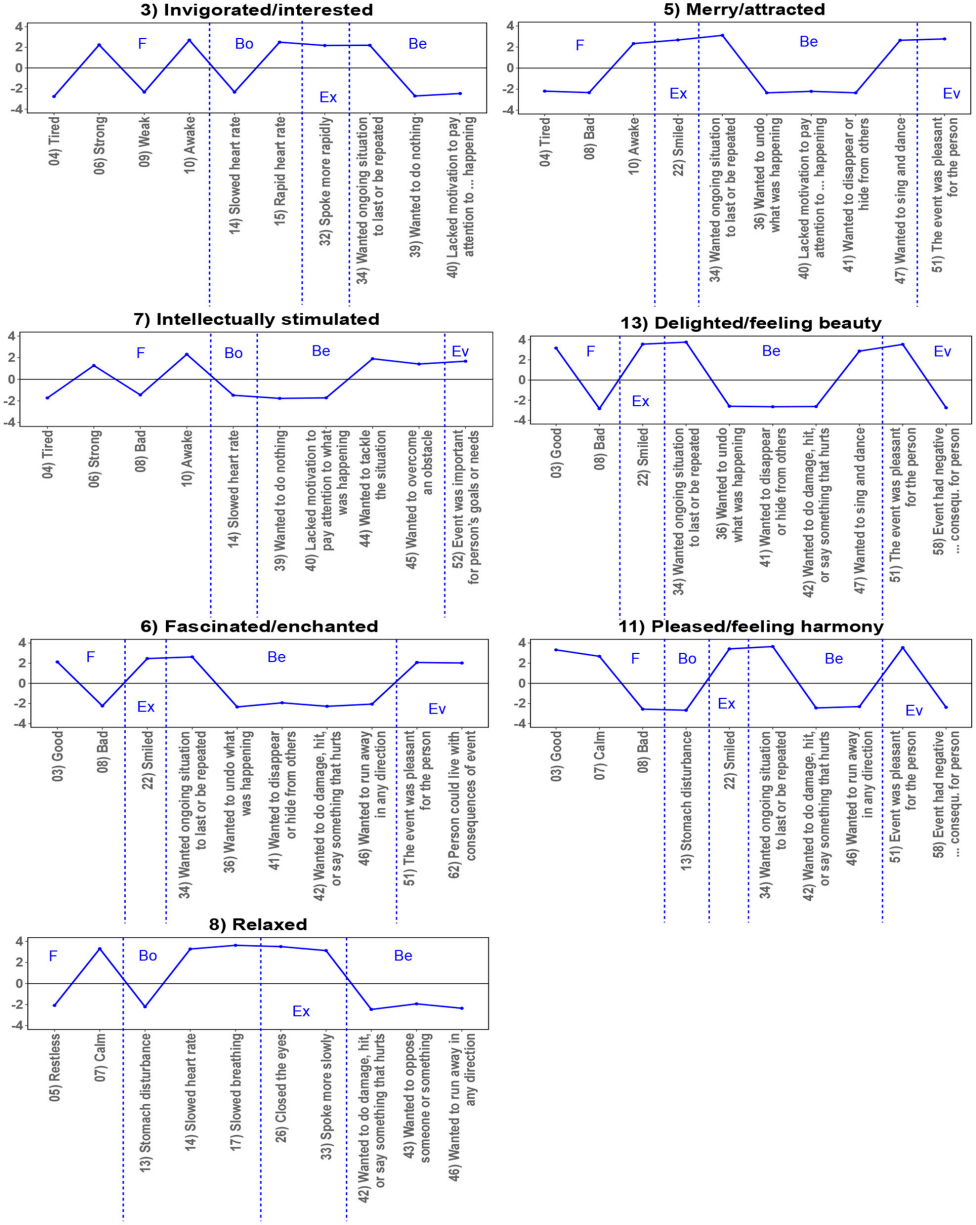
Profiles displaying the top and bottom 5 features for the custers indicating (rather) positive valence, allocated approximately according to their position in the right half of Figure 2. Features are partly abbreviated and can be read in full in Supplementary Tables 1, 2. F, Subjective Feeling; Bo, Bodily Reactions; Ex, Expression; Be, Behavior Tendencies; Ev, Event Evaluation. The figure has been generated using the R-packages *ggplot2* (Wickham, 2016) and svglite (Wickham et al., 2020) in RStudio (RStudio Team, 2019) and the open source vector graphics editor Inkscape (Bah, 2020).

To determine the features that are particularly characteristic for each cluster, we identified the five GRID features with the highest scores and the five GRID features with the lowest scores for each cluster. These are illustrated in Figures 3 and 4. The top and bottom features that are characteristic for each cluster support the interpretation mentioned above. Clusters on the negative end of valence typically included low cluster center scores of wishing for a situation to continue or be repeated, of appraisals of the pleasantness of an event, and of smiling; in contrast, these features were high for the positive end of the dimension. Concerning dimension 2, clusters on the high end of arousal had particularly low center scores for being tired or calm and high ratings for being awake, whereas the low pole was rather characterized by high scores of being tired or lacking the motivation to pay attention to what was going on.

Again, the clusters in the middle of both dimensions revealed a mixture of GRID features indicating either both positive and negative valence at the same time or none of them (such as having tears in the eyes, but without an accompanying negative (sadness) nor positive (e.g., tears of happiness) subjective feeling) and features neither indicative of particularly high or low arousal. It follows that valence and arousal alone cannot sufficiently account for some of the clusters. This applies specifically to those clusters which are sometimes referred to as prototypical aesthetic emotions (e.g., Schindler et al., 2017).

Some features seem to play only a minor role in distinguishing the cluster. Thus, the appraisal of an event as relevant to the person's goals or needs was among the top or bottom five features only once (high center score in Cluster 7 “intellectually stimulated”), and the relevance of an event to goals or needs of *somebody else* did not at all appear among the top or bottom five features of any cluster. A more detailed description of the results can be found in the Supplementary Material.

## Discussion

In the current study, we pursued two aims: (1) To examine the dimensionality of the GRID features when applied to aesthetic emotions, and (2) to identify clusters that might further illuminate the internal organization of aesthetic emotion terms.

### Dimensions Underlying the Semantic Meaning of Aesthetic Emotions

One of the aims in this study was to determine the nature of the semantic space for the selected sample of major aesthetic emotions, in particular the number and nature of the dimensions required to capture the differentiation between the terms with a reasonably high level of the variance explained. While earlier research suggested three dimensions (valence, arousal, power; Osgood et al., 1957; Shaver et al., 1987), others (e.g., Russell, 1980; Watson and Tellegen, 1985; Feldman, 1995) claimed that two dimensions (valence, arousal) might be sufficient. As mentioned in the introduction, the recent work on the semantic space of emotion terms, employing an appropriate feature-based approach across a large number of languages worldwide, has demonstrated that four dimensions (valence, arousal, power, novelty; Fontaine et al., 2007, 2013, 2021; Scherer et al., 2013; Gillioz et al., 2016; Gentsch et al., 2018) are required to differentiate the emotion terms in a satisfactory fashion.

Based on the results of the current study with the Aesthetic Emotion GRID data, a three to five-dimensional space can be extracted, consisting of valence, arousal, and power in all three solutions (see Osgood et al., 1957; Shaver et al., 1987), and additionally of novelty in the four- and five-dimensional solutions. In the five-dimensional solution, the power dimension is divided into two subdimensions (power motivation and power potential; see Supplementary Tables 3, 4). It has been argued that one major difference between aesthetic and utilitarian emotions concerns the appraisals of goal relevance and coping potential (Scherer and Zentner, 2008; Scherer and Coutinho, 2013; Lajante et al., 2020). Typically, aesthetically perceived objects or events do not have major implications for our goals in daily life or for our survival. However, the role of novelty has been emphasized in particular for the domain of aesthetic emotions (Fayn et al., 2015; Menninghaus et al., 2019). In consequence, we settled on a four-dimensional solution representing the dimensions valence, power, arousal, and novelty. This corresponds to the interpretation and sequence of dimensions in other studies using the GRID paradigm (Fontaine et al., 2007, 2021; Scherer et al., 2013; Gillioz et al., 2016; Gentsch et al., 2018).

The first dimension—readily interpretable as *valence*—was especially represented by behavioral tendencies such as wanting a situation to be continued or repeated, and appraisals referring to the (in-)consistency of an event with a person's standards—features that had been found to load on the valence dimension in prior studies as well. The aesthetic emotion terms ranged from feelings of beauty, enchantment and perfection to feelings of discomfort, repulsion and dislike, supporting the notion of aesthetic emotions as involving an evaluation of the aesthetic experience (Menninghaus et al., 2019).

Again in accordance to prior GRID studies, *power* emerged as the second dimension—a dimension that was already proposed in the 1950ies (Osgood et al., 1957; Shaver et al., 1987). The interpretation of the power dimension is unambiguous. The attribution of the features “spoke with a trembling voice” or “had tears in the eyes” (representing [low] power) is supported by other GRID studies (see Gillioz et al., 2016; Fontaine et al., 2021). Furthermore, features such as “The person was powerless in this situation involving the event” or “The person had power over the consequences of the event,” while showing relatively high cross loadings on valence, loaded on power. Notably, these latter features loaded highest on valence in the studies by Fontaine et al. (2021) and Gillioz et al. (2016). The explanation seems straightforward: having power feels good, which might be the reason for both the cross-loadings in the current data and the main loadings of these features on valence in other studies (see also Fontaine et al., 2013). The aesthetic emotion terms ranged from feeling humbled and overwhelmed to feelings of intellectual challenge, but also boredom. Whereas most terms on the low pole of power are readily interpretable, the interpretation of boredom in terms of high power may not seem straightforward. However, feeling bored by an aesthetically evaluated object infers a judgmental stance: by feeling bored, an observer positions himself “above” an artist, thus empowering him/herself with the power of (negative) judgment (Moller, 2014). In contrast, positive evaluations often imply a sense of a superior power on the part of an artist and can—-particularly in the case of admiration—-tend to dwarf the observer.

One has to consider that aesthetically perceived events (such as listening to an opera or watching a theater play) might be of much lower consequence to individuals when they watch scenes acted out on the stage as contrasted to when they are actively involved in or impacted by a similar event. This passive stance will obviously render an appraisal of control, power, or coping potential much less salient. In an attempt to distinguish aesthetic from other types of emotions, Frijda (1989) point out the reality aspect: emotions elicited by aesthetically perceived events such as art or music or films can be as intense as emotions felt in an everyday event; yet, they are different from everyday events by virtue of referring to imagination. In Frijda's words, “No one jumps up to warn Jane of the approaching snake or warns Hamlet that Polonius is eavesdropping during his conversation with Ophelia” (Frijda, 1989, p. 1,546). Likewise, watching a movie scene that shows someone standing very close to the rim of a cliff might elicit feelings of fear particularly in someone afraid of heights. Yet, viewers know they cannot actually fall down that cliff—as they are comfortably sitting in a chair in their living room or the movie theater—they know they are safe. Thus, while lacking any coping potential in the depicted situation, there are no negative consequences for one's own safety, making the coping potential less relevant while leaving the negative valorization intact.

This interpretation is also in accord with the distinction of utilitarian and aesthetic emotions provided by Coutinho and Scherer (2017). Utilitarian emotions entail evaluations important for the adjustment to events with important consequences for survival, whereas aesthetic emotions refer to evaluations of stimuli in terms of their intrinsic qualities, resulting in emotions such as awe, being moved or also being bored. The reality aspect sets aesthetic emotions also apart from achievement emotions (Gentsch et al., 2018). Failing an exam, for example, is likely to have more dire consequences for an individual than feeling tension or fear when watching a movie. Thus, the power dimension is well-reflected by its features and emotion terms and largely converges with former GRID studies.

Many features loading on the third dimension *arousal* are comparable to prior GRID studies, such as faster or slower breathing, or rapid and slowed heartrate (Fontaine et al., 2013, 2021; Gillioz et al., 2016; Gentsch et al., 2018). Furthermore, features such as “wanted to do nothing” and “tired” loaded on arousal (features that had been found to load on power and valence, respectively; Gillioz et al., 2016). As is the case for the power dimension, the typical role of a person as an observer rather than an active participant of an event (Frijda, 1989) might affect the arousal aspect of behavioral tendencies and subjective feelings. Arousal or emotional activation has been central to aesthetic perception already since Berlyne (1971) developed the psychobiological model of aesthetics. As argued by Menninghaus et al. (2019), aesthetic emotions can span the full range from low to high arousal, for example feelings of peacefulness vs. feelings of suspense, excitement, or even anger. This is evident in the emotion terms that stretch from feelings of energy and enthusiasm to relaxation and boredom (Figure 1B). Thus, the dimension *arousal* identified in the current data set is in accordance with existing literature on aesthetic perceptions and emotions.

*Novelty* emerged as a fourth dimension. Features loading on novelty included both features from the event evaluation component (“The event occurred suddenly,” “The event was unpredictable”) as well as the expression component (“Raised the eyebrows,” “Dropped their jaw,” the latter being the prototypical facial appearances expressing surprise; Ekman, 1992; Scherer et al., 2018). Novelty plays an important role in Scherer's (1984, 2005, 2009) Component Process Model of emotions in general (as part of the relevance appraisal of an event). Furthermore, among others, Menninghaus et al. (2019) considered novelty as crucial for aesthetic emotions and argued that novelty in the domain of aesthetic emotions differs from novelty in everyday emotions by representing something categorically innovative, rather than novelty concerning the current situation one might find him/herself in. In the domain of aesthetic emotions, novelty and complexity, as long as they are not cognitively over-demanding for individuals, might trigger interest in the stimulus or event (Silvia, 2010; Fayn et al., 2015). Accordingly, feelings of surprise, followed by feelings of curiosity and sparked interest, are represented on the higher end of novelty. Thus, in sum, although both three- and five-dimensional solutions might be feasible, the four dimensions identified by previous GRID studies (Fontaine et al., 2007, 2013, 2021; Gillioz et al., 2016; Gentsch et al., 2018) are well-represented by the respective GRID features for the aesthetic emotion terms investigated in the current study. There are only slight differences with respect to the salience of certain features found in the earlier GRID studies of general emotion terms.

### Internal Organization of the Domain of Aesthetic Emotions: Identification of Clusters

We derived 15 clusters from the semantic feature profiles of 75 emotion terms as determined specifically for the context of aesthetics. These clusters can be interpreted as revealing how lay people organize aesthetic emotions into “families” based on similarities of underlying features. Several of the cluster findings are in line with a recent theoretical conceptualization of aesthetic emotions according to which these emotions make a direct contribution to aesthetic evaluation (Menninghaus et al., 2019; see also Fingerhut and Prinz, 2020). The Aesthetic Emotions GRID captures a major behavior tendency that is reflective of aesthetic evaluation: to seek (or not to seek) continued and repeated exposure to the emotion-eliciting situation (see Menninghaus et al., 2019, 2020). For ten out of 15 clusters (see Figures 3, 4), this behavior tendency was among the five GRID features with the five highest (1 “moved/in awe,” 3 “invigorated/interested,” 5 “merry/attracted,” 6 “fascinated/enchanted,” 11 “pleased/feeling harmony,” 13 “delighted/feeling beauty”) or, respectively, lowest (4 “confused/worried,” 10 “sad,” 12 “displeased/repelled,” 14 “bored”) center scores. Thus, it emerged as one of the most relevant features within the context of aesthetics.

Two other features were distinctive in more than half of the clusters: the facial expression of smiling together with the evaluation of the event as pleasant for the person (4 clusters with high scores on both features: 5 “merry/attracted,” 6 “fascinated/enchanted,” 11 “pleased/feeling harmony,” 13 “delighted/feeling beauty;” 4 clusters with low scores on both features: 4 “confused/worried,” 10 “sad,” 12 “displeased/repelled,” 15 “angry”). This provides further support for the preeminent importance of intrinsic pleasantness appraisals to aesthetic experience.

However, the cluster findings also underscore that there are types of aesthetic appeal that cannot be reduced to intrinsic pleasantness and positive valence. Specifically, the emotions in clusters 1 “moved/in awe,” 3 “invigorated/interested,” and 7 “intellectually stimulated” typically were not rated as markedly pleasant. Rather, our respondents associated these emotions with high levels of attentiveness, as revealed by the features “feeling awake” rather than “tired” and not “lacking the motivation to pay attention to what was happening.” The emotions in these clusters may include some negative ingredients, which according to the Distancing-Embracing model (Menninghaus et al., 2017) are particularly suited to secure attention to, emotional engagement in, and memorability of an aesthetic experience.

It is also informative to examine which GRID features did not differentiate well between the clusters. Thus, evaluating the event as important for and relevant to the person's goals or needs appeared in Figures 3 and 4 only once; this underscores that pragmatic interest and goal relevance are of minor importance to distinguishing between clusters of aesthetic emotions (see Menninghaus et al., 2019). Even for cluster 7 “intellectually stimulated,” for which this appraisal was characteristic, we can assume that the relevant goal was to understand and to derive meaning from the aesthetic experience, but not any pragmatic benefit. The associated motivation to tackle the situation and to overcome an obstacle is suggestive of a complex experience that—depending on one's success with decoding its meaning—can lead to insight and heightened interest and/or confusion (Silvia, 2009; Fayn et al., 2019). Confusion, while it may be positively associated with interest (for instance in people high in openness to experience; Fayn et al., 2019), usually implies a negative evaluation of the eliciting situation.

This is supported by our cluster findings, which show a clear separation between clusters of emotion terms that are used to characterize positively valued aesthetic experiences, and those that are used to imply low aesthetic value. Cluster 14 “bored” stands out as aversive due to a lack of arousing or attention-grabbing features of the situation or event. As stated by Moller (2014), “calling art boring is among the very worst of insults” (p. 183).

In contrast, negative basic emotions have been considered as emotions that make a positive contribution to aesthetic liking, as people—at least sometimes—seem to take pleasure in negative affect (Menninghaus et al., 2017). For example, people feel entertained by horror movies (Bartsch et al., 2010); they enjoy feelings of nostalgia and melancholy when listening to sad music (Silvia, 2009; Bartsch, 2012; Taruffi and Koelsch, 2014; Hosoya et al., 2017), or delight in disgusting objects in paintings. For these reasons, one aim when developing the AESTHEMOS scale had been to include both positive and negative emotions as well as emotions which involve both positive and negative ingredients (Schindler et al., 2017). Our respondents' ratings show that they do not enjoy negative emotions as such. Rather, they conceptualized negative basic emotion terms as indicating an aversive rather than positive aesthetic experience. Even when explicitly asked about experiences with aesthetically perceived objects and events, they did not think that the semantic meaning of sadness, fear, or anger would change to indicate a pleasurable experience. Rather, negative emotion labels were thought to be used for events that are highly inconsistent with the person's own standards and ideals and, thus, creating antagonism and action tendencies of rejection or distancing (Silvia and Brown, 2007; Silvia, 2009).

Thus, while atypical variants of negative emotions (e.g., pleasant fear, pleasant sadness; see Wilson-Mendenhall et al., 2015) can occur within the context of aesthetics, people most likely would not spontaneously choose to label these emotions as fear or, respectively, sadness. Rather, they tend to choose emotion labels that are specialized in expressing some negativity of eliciting events and stimuli along with inherent reward dimensions that primarily support the approach motivation, such as being sadly moved (Menninghaus et al., 2015; Vuoskoski and Eerola, 2017), morbid fascination (Rimé et al., 2005; Oosterwijk et al., 2016), or threat-based awe (Gordon et al., 2017). Labels such as moved, fascinated, and awe are generally indicative of liking, but they may nevertheless capture feelings of sadness and fear and, thus, account for indirect effects of negative emotions on aesthetic appreciation (Menninghaus et al., 2019, 2020).

### Implications for the Understanding of Aesthetic Emotions and for Future Research

Using the GRID paradigm, the same four dimensions have been found for a large number of languages. While the findings of the current study are exploratory, together with the study by Gentsch et al. (2018), the current study provides evidence that the same dimensionality can be identified in other domains of emotions as well. A close look on the top loading features in the current data as compared to other studies (e.g., Gillioz et al., 2016; Fontaine et al., 2021) revealed subtle differences regarding the apparent salience of features in aesthetic emotions. For example, for the valence dimension, a shift from features that focus on the person (smiling, pleasant, feeling good) toward features that rather focus on the event in question (which should continue or be repeated or which is inconsistent with one's standards) can be noted in the Aesthetic Emotions GRID data. However, the loading differences are sometimes minute, so that further investigations are needed before any reliable conclusions are warranted. Furthermore, it might be worthwhile to examine whether the dimensionality reported here for German emotion terms applies to the domain of aesthetic emotions in other languages and cultures as well in order to see whether the results obtained here are generalizable.

The present findings are informative for future research on aesthetic emotions, as they provide insight into the semantic meaning that respondents attach to specific aesthetic emotion terms. We have used the same set of emotion labels in two other studies. One study examined self-reported emotional experiences after a broad range of aesthetically perceived events (Schindler et al., 2017), and in the other study, participants sorted the emotion labels into piles, based on perceived similarities between the labels (Hosoya et al., 2017). As would be expected based on the different methods used, the findings of the three studies show some differences when comparing them in every detail. Nevertheless, all three studies converge in suggesting that participants consistently conceptualize and actually experience aesthetic emotions as reflective of different kinds of aesthetic virtues or vices. All three studies also demarcate a broad group of negative emotions that typically characterize disliked aesthetic experiences; these include all negative basic emotions along with boredom, confusion, and feelings of ugliness and repulsion.

Furthermore, all three studies show a great range of emotions that are associated with positive aesthetic evaluation: clearly, there is not just one kind of aesthetic pleasure or gratification (Bartsch and Viehoff, 2010; Menninghaus et al., 2019; Fingerhut and Prinz, 2020; Diessner et al., 2021). Rather, it is possible to distinguish epistemic emotions which are associated with pleasurable cognitive engagement, prototypical, and often mixed aesthetic emotions which are highly captivating, and pleasing emotions ranging from the good feeling of exhilaration to peaceful relaxation. Taken together, the clusters found in all three studies could serve as a basis for the development of a short scale that may lend itself for use in field studies on aesthetic emotions.

### Limitations

Some limitations have to be considered. In the present study, the semantic structure and organization of the domain of aesthetic emotions were investigated. Some of the terms, especially the ones considered as prototypical aesthetic emotions (e.g., nostalgia, longing, awe), seem to be more difficult to grasp than other emotion terms, especially those that are more commonly used in everyday language (such as happy, calm, or pleasant), as indicated by the lower inter-rater consistencies (Supplementary Table 1). Combined with the rather complex GRID design, the inherent difficulty of the less commonly used terms may have affected the results.

In the current study, the dimensionality of the semantic space in the domain of aesthetic emotions was investigated for the first time. Therefore, an exploratory approach was used in order to investigate the number and nature of dimensions required to sufficiently and meaningfully describe aesthetic emotions. Based on different methods of determining the number of dimensions to be extracted, three- to five-dimensional structures are feasible. However, a three-dimensional solution lacks the novelty dimension which has been shown to be particularly relevant for aesthetic emotions. A five-dimensional solution, which explains only about 2% additional variance as compared to the four-dimensional solution, splits the power dimension into two subdimensions, which is of little consequence for aesthetic emotions. In consequence, we adopted the four-dimensional solution that corresponds to the solution found in earlier studies with respect to both the type and the extraction sequence of the dimensions (Fontaine et al., 2007, 2021; Fontaine and Veirman, 2013; Scherer et al., 2013; Gillioz et al., 2016; Gentsch et al., 2018). However, the results obtained in the current study were obtained only for the German language limiting the generalizability of the results and requiring replication in future studies, including work across other cultures and languages, before a firm conclusion on the dimensionality can be drawn.

Lastly, the instructions for the task mentioned examples for possible aesthetic experiences; the list is not an exhaustive list of possible events, but intended to give participants an impression of possible situations that could lead to experiencing an aesthetic emotion term such as “was enthusiastic.” As one example of an aesthetic experience in nature, a sunset was mentioned in order to cover a range of aesthetic experiences including nature. However, experiences in nature may have real and possibly serious consequences (e.g., a thunderstorm), while watching such scenes in a theater play or in television, or visiting a museum, do not. Thus, they may differ in respect to the reality aspect of the aesthetically perceived events (Frijda, 1989). Including nature examples with potential danger may have led to different results for example concerning the power dimension. However, most descriptions of beauty in nature (such as the sunset mentioned in the instruction) do not involve any real danger, but are readily combined with feelings of “harmony” and being in peaceful sync with nature. The limitation is limited to the sublime and awe in experiencing nature and hence to a sub-segment only.

### Conclusion

In accordance with prior studies (e.g., Fontaine et al., 2013, 2021; Gillioz et al., 2016; Gentsch et al., 2018), the data of the current study support a four-dimensional space covering valence, power, arousal, and novelty. While the results are exploratory, these findings indicate that this four-dimensional space is not restricted to everyday emotions, but equally applies to the domain of aesthetic emotions. Furthermore, clusters were identified that shed light on how classes of aesthetic emotions are understood by laypeople. The findings show considerable convergence with former studies addressing the same aesthetic emotion terms while using different methodologies (Hosoya et al., 2017; Schindler et al., 2017). This clearly strengthens the findings both of the present and the earlier studies.

In conclusion, this study elucidates the semantic structure and representation of a particular subgroup of emotions, namely aesthetic emotions. It provides a solid basis for further research and methodological development, such as the investigation of the dimensionality and clusters of aesthetic emotions in other languages and cultures or the construction of a brief aesthetic emotions rating scale for use in field studies of aesthetic experiences.

## Data Availability Statement

The dataset and analyses script presented in this study can be found in online repositories. The names of the repository/repositories and accession number(s) can be found at: https://osf.io/8f6em/ under the name “Dimensions and Clusters of Aesthetic Emotions” (uploaded by Ursula Beermann).

## Ethics Statement

The study was part of a larger research project (see Funding information) for which the Ethics Committee of the University of Geneva, the host of the project, approved the research procedures. Participation was voluntary and anonymous. The participants gave informed consent by clicking on the next button after receiving information about the study. They were free to withdraw their consent to participate at any time.

## Author Contributions

KRS acquired funding and designed the overall project PROPEREMO. All authors designed the study described in this manuscript. UB implemented the task in the GRID study interface. GH and IS supervised the data collection. UB and GH analyzed the data. UB wrote the first draft of the manuscript, with contributions of IS in the introduction and discussion section, and GH in the analyses description and the result section of the cluster analyses. All authors provided feedback to the manuscript. UB and KRS finalized the manuscript.

## Conflict of Interest

The authors declare that the research was conducted in the absence of any commercial or financial relationships that could be construed as a potential conflict of interest.
