# Supplementary material for: Dimensions and Clusters of Aesthetic Emotions: A Semantic Profile Analysis
Source: Front Psychol. 2021 May 28;12:667173. doi: 10.3389/fpsyg.2021.667173 (PMC8194692; doi:10.3389/fpsyg.2021.667173)
Supplement: Supplementary file 1 [file Data_Sheet_1.pdf]

Dimensions and Clusters of Aesthetic Emotions: A Semantic Profile Analysis

Supplementary Material

Ursula Beermann[1], Georg Hosoya[2], Ines Schindler[3], Klaus R. Scherer[4,5*] , Michael Eid[2], Valentin Wagner[3,6], and Winfried Menninghaus[3]

[1]Department of Psychology, UMIT - Private University for Health Sciences, Medical Informatics and Technology, Hall in Tirol, Austria.
[2]Department of Education and Psychology, Division of Methods and Evaluation, Freie Universität Berlin, Berlin, Germany.
[3] Max Planck Institute for Empirical Aesthetics, Frankfurt am Main, Germany.
[4] Department of Psychology, University of Geneva, Geneva, Switzerland.
[5] Department of Psychology, Ludwig-Maximilians-University Munich, Munich, Germany.
[6] Humanities and Social Sciences, Helmut Schmidt University/University of the Federal Armed Forces Hamburg, Hamburg, Germany.

Supplementary Table 1

Inter-rater Consistency (Cronbach α) for Each of the 75 Emotion Terms Across the CoreGRID Features (Uncentered Scores), Sorted From Highest to Lowest α Coefficient

Nr.	German	English	Group	α_C	N_C	α_A	N_A
7	Beruhigte mich	Calmed me	11	.959	15	.959	15
55	Empfand ich als angenehm	I found it pleasant	7	.959	15	.959	15
10	Machte mich zufrieden	Made me feel content	5	.957	16	.957	16
11	Machte mich glücklich	Made me happy	6	.957	16	.957	16
4	Stimmte mich fröhlich	Made me cheerful	2	.956	17	.956	17
13	Erfreute mich	Delighted me	9	.955	15	.955	15
30	Begeisterte mich	Made me feel enthusiastic	11	.954	14	.946	15
39	Energetisierte mich	Energized me	6	.952	16	.952	16
56	Beängstigte mich	Scared me	7	.951	15	.951	15
74	Entspannte mich	Relaxed me	8	.950	16	.950	16
28	Empfand ich als schön	I found it beautiful	4	.949	16	.949	16
26	Schockierte mich	Was shocking to me	2	.946	17	.946	17
8	Stimmte mich ärgerlich	Made me angry	11	.945	14	.936	15
35	Machte mich aggressiv	Made me aggressive	4	.944	16	.944	16
37	Fühlte etwas Wunderbares	Felt something wonderful	4	.944	16	.934	16
25	Gefiel mir	Liked it	3	.942	16	.942	16
61	Fühlte mich verwirrt	Felt confused	5	.942	15	.924	16
5	Wirkte auf mich beklemmend	Felt oppressive	11	.941	14	.933	15
52	Machte mich munter	Perked me up	7	.941	14	.929	15
71	Amüsierte mich	Amused me	9	.937	15	.937	15
14	Empfand ich als vollkommen	I found it perfect	11	.935	14	.927	15
47	Wühlte mich auf	Agitated me	4	.935	16	.935	16
44	Überraschte mich	Surprised me	7	.933	14	.921	15
72	Empfand ich als harmonisch	I found it harmonious	10	.932	15	.932	15
16	Beunruhigte mich	Worried me	10	.931	15	.931	15
34	Machte mich neugierig	Made me curious	11	.931	15	.931	15
63	Empfand ich als geschmacklos	I found it distasteful	9	.930	15	.930	15
2	Beflügelte mich	Spurred me on	3	.929	16	.929	16
54	Fühlte mich in der Erfahrung aufgehen	Felt absorbed in the experience	5	.929	15	.919	16
42	Inspirierte mich	Inspired me	5	.928	15	.919	16
22	Fühlte mich bedrückt	Felt depressed	4	.927	15	.923	16

Nr.	German	English	Group	α_C	N_C	α_A	N_A
60	Empfand ich als unangenehm	I found it unpleasant	9	.927	15	.927	15
27	Stieß mich ab	Repelled me	2	.926	17	.926	17
3	Belebte mich	Invigorated me	10	.923	15	.923	15
40	Irritierte mich	Irritated me	7	.923	15	.923	15
64	War mir gleichgültig	Felt indifferent	4	.923	16	.923	16
21	Behagte mir nicht	Made me feel uncomfortable	3	.920	12	.882	16
53	Weckte meinen Tatendrang	Motivated me to act	9	.920	15	.920	15
70	Versetzte mich in träumerische Stimmung	Put me in a dreamy mood	9	.917	15	.917	15
59	Belustigte mich	Was funny to me	6	.916	16	.916	16
69	Verstörte mich	Was unsettling to me	8	.916	15	.905	16
43	Erheiterte mich	Made me merry	3	.915	15	.909	16
46	Weckte mein Interesse	Sparked my interest	4	.915	16	.915	16
50	War wie verzaubert	Was enchanted	7	.913	15	.913	15
29	Zog mich an	Was attracted	6	.912	16	.912	16
19	War beeindruckt	Was impressed	6	.910	16	.910	16
31	Verblüffte mich	Baffled me	4	.906	15	.900	16
67	Stimmte mich traurig	Made me sad	10	.906	15	.906	15
38	Ermüdete mich	Tired me	11	.905	15	.905	15
68	Langweilte mich	Bored me	7	.899	15	.899	15
6	Packte mich	Gripped me	3	.888	15	.882	16
9	Empfand ich als erhaben	I found it sublime	2	.887	15	.862	17
49	War überwältigt	Was overwhelmed	9	.886	13	.864	15
36	Missfiel mir	Disliked it	6	.885	16	.885	16
75	Faszinierte mich	Fascinated me	8	.884	15	.881	16
15	War ergriffen	Felt deeply moved	5	.876	14	.834	16
65	War hingerissen	Was enraptured	10	.872	13	.809	15
58	Empfand ich als hässlich	I found it ugly	6	.871	15	.867	16
32	Empfand Demut	Felt humbled	5	.869	13	.799	16
45	Spürte einen tieferen Sinn	Sensed a deeper meaning	8	.858	13	.835	16
48	Empfand ich als anmutig	I found it graceful	8	.857	14	.85	16
57	Versetzte mich in Staunen	Astonished me	10	.855	14	.841	15
17	Forderte mich intellektuell heraus	Challenged me intellectually	2	.854	15	.833	17
12	Berührte mich	Touched me	2	.853	16	.846	17
24	War geistig gefordert	Was mentally engaged	5	.851	14	.811	16

Nr.	German	English	Group	α_C	N_C	α_A	N_A
20	Stimmte mich melancholisch	Made me feel melancholic	3	.839	15	.83	16
18	War mir meiner selbst nicht bewusst	Was not aware of myself	2	.833	11	.736	17
73	Regte meine Gedanken an	Stimulated my thoughts	9	.832	13	.799	15
62	Spürte die Zeit wie im Flug vergehen	Felt that time is flying	8	.826	14	.787	16
66	Bewegte mich	Moved me	8	.790	15	.775	16
23	Fühlte eine plötzliche Einsicht	Felt a sudden insight	2	.789	14	.734	17
41	Weckte in mir sentimentale Gefühle	Made me feel sentimental	10	.787	13	.768	15
51	Empfand Ehrfurcht	Felt awe	8	.757	12	.659	16
33	Machte mich nostalgisch	Made me feel nostalgic	6	.739	13	.721	16
1	Weckte in mir Sehnsucht	Filled me with longing	3	.719	12	.667	16

Note. Group = Emotion group in which the phrase was presented within the study. N_C = Sample size of converging raters; N_A = Sample size of all raters; α_C = Cronbach's Alpha across only converging raters (participant-rest correlation ≥ .20); α_A = Cronbach's Alpha across all raters (see Fontaine et al., 2013).

Supplementary Table 2

German Features, their English Translations and the Respective Component of Each Feature according to the CPM (Scherer, 2013)

Nr.	Feature (German)	Feature (English)	Component
3	Gut	Good	Feeling
4	Müde	Tired	Feeling
5	Unruhig	Restless	Feeling
6	Stark	Strong	Feeling
7	Ruhig	Calm	Feeling
8	Schlecht	Bad	Feeling
9	Schwach	Weak	Feeling
10	Wach	Awake	Feeling
11	Sich schwach in den Gliedern fühlen	Feeling weak in the limbs	Body
12	Blass werden	Becoming pale	Body
13	Magenbeschwerden	Stomach disturbance	Body
14	Verlangsamter Herzschlag	Slowed heart rate	Body
15	Beschleunigter Herzschlag	Rapid heart rate	Body
16	Muskelanspannung	Tense muscles	Body
17	Verlangsamte Atmung	Slowed breathing	Body
18	Beschleunigte Atmung	Rapid breathing	Body
19	Sich warm fühlen	Feeling warm	Body
20	Schwitzen	Sweating	Body
21	Sich kalt fühlen	Feeling cold	Body
22	Lächelte	Smiled	Expression
23	Die Kinnlade fallen ließ	Dropped their jaw	Expression
24	Die Augenbrauen hoch zog	Raised the eyebrows	Expression
25	Die Stirn runzelte	Frowned	Expression

Nr.	Feature (German)	Feature (English)	Component
26	Die Augen schloss	Closed the eyes	Expression
27	Tränen in den Augen hatte	Had tears in the eyes	Expression
28	Lauter sprach	Spoke more loudly	Expression
29	Mit zitternder Stimme sprach	Spoke in a trembling voice	Expression
30	Mit fester Stimme sprach	Spoke in a firm voice	Expression
31	Sprechprobleme hatte	Had speech disturbances	Expression
32	Langsamer sprach	Spoke more slowly	Expression
33	Schneller sprach	Spoke more rapidly	Expression
34	Wollte, dass die Situation weiter andauert oder sich wiederholt	Wanted the ongoing situation to last or be repeated	Behavior
35	Beenden wollte, was er/sie gerade tat	Wanted to stop what he/she was doing	Behavior
36	Das, was gerade passierte, rückgängig machen wollte	Wanted to undo what was happening	Behavior
37	Sich nach den Wünschen von jemand anderem richten wollte	Wanted to comply with someone else's wishes	Behavior
38	Die Initiative jemand anderem überlassen wollte	Wanted someone else to take the initiative	Behavior
39	Nichts machen wollte	Wanted to do nothing	Behavior
40	Keine Lust hatte, die Aufmerksamkeit auf das zu richten, was gerade passierte	Lacked the motivation to pay attention to what was happening	Behavior
41	Verschwinden oder sich vor anderen verstecken wollte	Wanted to disappear or hide from others	Behavior
42	Schaden anrichten, zuschlagen oder etwas Verletzendes sagen wollte	Wanted to do damage, hit, or say something that hurts	Behavior
43	Sich jemandem oder etwas widersetzen wollte	Wanted to oppose someone or something	Behavior
44	Die Situation meistern wollte	Wanted to tackle the situation	Behavior
45	Ein Hindernis überwinden wollte	Wanted to overcome an obstacle	Behavior
46	In irgendeine Richtung wegrennen wollte	Wanted to run away in any direction	Behavior
47	Singen oder tanzen wollte	Wanted to sing and dance	Behavior

Nr.	Feature (German)	Feature (English)	Component
48	Das Ereignis plötzlich auftrat	The event occurred suddenly	Evaluation
49	Das Ereignis unvorhersehbar war	The event was unpredictable	Evaluation
50	Das Ereignis die Erwartungen der Person bestätigte	The event confirmed the expectations of the person	Evaluation
51	Das Ereignis angenehm für die Person war	The event was pleasant for the person	Evaluation
52	Das Ereignis wichtig und relevant für die Ziele und Bedürfnisse der Person war	The event was important for and relevant to the person's goals or needs	Evaluation
53	Das Ereignis wichtig und relevant für die Ziele und Bedürfnisse von jemand anderem war	The event was important for and relevant to the goals or needs of somebody else	Evaluation
54	Das Ereignis zufällig passierte	The event happened by chance	Evaluation
55	Das Ereignis durch das eigene Verhalten der Person verursacht wurde	The event was caused by the person's own behavior	Evaluation
56	Das Ereignis durch das Verhalten von jemand anderem verursacht wurde	The event was caused by somebody else's behavior	Evaluation
57	Das Ereignis vorhersehbare Konsequenzen hatte	The event had consequences that were predictable	Evaluation
58	Das Ereignis negative, unerwünschte Konsequenzen für die Person hatte	The event had negative, undesirable consequences for the person	Evaluation
59	Das Ereignis sofortiges Handeln erforderte	The event required an immediate response	Evaluation
60	Die Person Macht über die Konsequenzen des Ereignisses hatte	The person had power over the consequences of the event	Evaluation
61	Die Person Kontrolle über die Konsequenzen des Ereignisses hatte	The person had control over the consequences of the event	Evaluation
62	Die Person mit den Konsequenzen des Ereignisses leben konnte	The person could live with the consequences of the event	Evaluation
63	Das Ereignis unvereinbar mit den Ansprüchen und Idealen der Person war	The event was inconsistent with the person's own standards and ideals	Evaluation

Nr.	Feature (German)	Feature (English)	Component
64	Das Ereignis eine Verletzung von sozial-akzeptierten Standards mit sich brachte	The event involved the violation of socially accepted norms	Evaluation
65	Die Person während dieses Ereignisses machtlos war	The person was powerless in this situation involving the event	Evaluation
66	Die Person eine dominante Position während dieses Ereignisses hatte	The person had a dominant role in the situation involving the event	Evaluation
67	Es keinen Grund zur Eile während dieses Ereignisses gab	There was no urgency in the situation involving the event	Evaluation
68	Das Ereignis unkontrollierbar war	The event was uncontrollable	Evaluation

Supplementary Table 3

Rotated Component Matrix of the GRID Features Resulting from a Principal Component Analyses Using Orthogonal Equamax Rotation, Extracting 3 Dimensions

Feature	Comp.	Comm.	Valence	Arousal	Power
Wanted the ongoing situation to last or be repeated	Be	.977	**-.904**	.011	-.399
The event was inconsistent with the person's own standards and ideals	Ev	.933	**.895**	.120	.342
Wanted to undo what was happening	Be	.942	**.882**	-.055	.402
The event was pleasant for the person	Ev	.977	**-.880**	-.067	-.445
Smiled	Ex	.951	**-.875**	-.044	-.429
Wanted to stop what he/she was doing	Be	.894	**.874**	-.167	.319
Good	F	.970	**-.873**	-.061	-.453
Wanted to do damage, hit, or say something that hurts	Be	.853	**.869**	.227	.214
The event had negative, undesirable consequences for the person	Ev	.938	**.867**	.088	.422
Bad	F	.944	**.866**	-.097	.430
Feeling warm	Bo	.897	**-.849**	-.067	-.415
Wanted to sing and dance	Be	.917	**-.844**	.109	-.440
Wanted to oppose someone or something	Be	.853	**.839**	.385	-.013
Wanted to run away in any direction	Be	.892	**.831**	.138	.428
Stomach disturbance	Bo	.939	**.819**	.078	.511
The event involved the violation of socially accepted norms	Ev	.840	**.806**	.273	.339
Frowned	Ex	.691	**.794**	.139	.204
Wanted to disappear or hide from others	Be	.912	**.776**	-.147	.537
Feeling cold	Bo	.894	**.771**	-.297	.460
The event was important for and relevant to the person's goals or needs	Ev	.791	**-.761**	.157	-.432
The person could live with the consequences of the event	Ev	.806	**-.711**	-.315	-.449
Restless	F	.859	**.710**	.487	.341

Feature	Comp.	Comm.	Valence	Arousal	Power
Strong	F	.907	**-.687**	.202	-.628
The event was important for and relevant to the goals or needs of somebody else	Ev	.617	**-.680**	.130	-.372
Lacked the motivation to pay attention to what was happening	Be	.803	**.665**	-.563	.208
There was no urgency in the situation involving the event	Ev	.862	**-.660**	-.583	-.294
Awake	F	.805	**-.569**	.483	-.498
Wanted to comply with someone else's wishes	Be	.550	**-.547**	-.499	-.026
Slowed heart rate	Bo	.929	-.205	**-.932**	-.141
Spoke more slowly	Ex	.894	-.138	**-.926**	.132
Rapid heart rate	Bo	.862	-.141	**.915**	.071
Slowed breathing	Bo	.891	-.258	**-.902**	-.104
Rapid breathing	Bo	.832	-.036	**.901**	.135
Wanted to do nothing	Be	.843	.039	**-.882**	.253
Tense muscles	Bo	.825	.249	**.870**	.072
Spoke more rapidly	Ex	.853	-.211	**.869**	-.233
Tired	F	.815	.275	**-.860**	.034
Closed the eyes	Ex	.779	-.228	**-.847**	.099
The event occurred suddenly	Ev	.748	-.181	**.817**	.218
Spoke more loudly	Ex	.785	-.133	**.788**	-.382
Sweating	Bo	.770	.338	**.784**	.202
The event was unpredictable	Ev	.697	.035	**.763**	.337
The event required an immediate response	Ev	.760	.457	**.730**	-.136
Calm	F	.909	-.568	**-.694**	-.325
Wanted someone else to take the initiative	Be	.688	.320	**-.647**	.409
Dropped their jaw	Ex	.518	-.167	**.639**	.285
Raised the eyebrows	Ex	.458	.205	**.632**	.128

Feature	Comp.	Comm.	Valence	Arousal	Power
The event happened by chance	Ev	.550	-.387	**.617**	.139
The event had consequences that were predictable	Ev	.637	-.236	**-.587**	-.487
Spoke in a firm voice	Ex	.871	-.406	.133	**-.829**
The person had a dominant role in the situation involving the event	Ev	.854	-.457	.013	**-.803**
Spoke in a trembling voice	Ex	.780	.268	.268	**.797**
Had speech disturbances	Ex	.788	.233	.371	**.772**
The person had control over the consequences of the event	Ev	.918	-.536	-.243	**-.756**
Feeling weak in the limbs	Bo	.879	.426	-.385	**.740**
The person had power over the consequences of the event	Ev	.854	-.536	-.158	**-.736**
The person was powerless in this situation involving the event	Ev	.853	.599	.057	**.701**
The event was caused by the person’s own behavior	Ev	.714	-.377	-.291	**-.698**
Becoming pale	Bo	.871	.639	-.006	**.680**
Wanted to overcome an obstacle	Be	.699	-.034	.556	**-.624**
The event was uncontrollable	Ev	.819	.503	.422	**.623**
Wanted to tackle the situation	Be	.777	-.474	.422	**-.612**
The event confirmed the expectations of the person	Ev	.853	-.563	-.423	**-.597**
Weak	F	.849	.581	-.412	**.584**
Had tears in the eyes	Ex	.386	-.295	-.040	**.545**
The event was caused by somebody else’s behavior	Ev	.335	.194	.323	**.439**

Note. Comp. = Component according to the CPM. Comm. = Communality. F=Subjective Feeling, Bo=Bodily Reactions, Ex=Expression, Be=Behavior Tendencies, Ev=Event Evaluation. Boldface indicates the highest loading of each feature. The feature loadings indicate that the two dimensions valence and power are inverted: for *valence*, positive loadings represent negative valence; for *power*, positive loadings stand for low power, and vice versa.

Supplementary Table 4

Rotated Component Matrix of the GRID Features Resulting from a Principal Component Analyses Using Orthogonal Equamax Rotation, Extracting 3 Dimensions

Feature	Comp.	Comm.	Valence	Arousal	Power Motivation	Power Potential	Novelty
Wanted to oppose someone or something	Be	.882	**.807**	.413	.198	.123	.072
Frowned	Ex	.887	**.760**	-.095	.296	.109	.449
Wanted to do damage, hit, or say something that hurts	Be	.856	**.750**	.242	.402	.250	.111
The event was inconsistent with the person's own standards and ideals	Ev	.935	**.744**	.131	.481	.350	.106
Wanted the ongoing situation to last or be repeated	Be	.978	**-.722**	.010	-.566	-.352	-.111
The event was pleasant for the person	Ev	.978	**-.716**	-.047	-.511	-.425	-.147
Smiled	Ex	.952	**-.713**	-.029	-.507	-.409	-.133
The event had negative, undesirable consequences for the person	Ev	.950	**.707**	.113	.484	.445	.072
Wanted to undo what was happening	Be	.945	**.706**	-.015	.540	.393	.025
Good	F	.972	**-.697**	-.065	-.526	-.437	-.114
Wanted to sing and dance	Be	.925	**-.684**	.143	-.522	-.386	-.121
Wanted to run away in any direction	Be	.923	**.680**	.177	.440	.483	.051
Feeling warm	Bo	.907	**-.679**	-.016	-.533	-.342	-.211
The event involved the violation of socially accepted norms	Ev	.841	**.677**	.226	.411	.330	.232
Wanted to stop what he/she was doing	Be	.907	**.668**	-.099	.621	.254	-.018
Bad	F	.953	**.657**	-.030	.602	.398	-.005
Wanted to comply with someone else's wishes	Be	.675	**-.647**	-.361	.124	-.192	-.273
Stomach disturbance	Bo	.947	**.642**	.085	.508	.507	.108

Feature	Comp.	Comm.	Valence	Arousal	Power Motivation	Power Potential	Novelty
Wanted to disappear or hide from others	Be	.921	**.592**	-.108	.530	.527	-.002
The person could live with the consequences of the event	Ev	.811	**-.586**	-.249	-.368	-.450	-.262
Feeling cold	Bo	.898	**.582**	-.281	.580	.381	.011
The event was important for and relevant to the goals or needs of somebody else	Ev	.629	**-.573**	.139	-.369	-.381	-.030
Restless	F	.871	**.558**	.466	.400	.335	.264
There was no urgency in the situation involving the event	Ev	.877	**-.558**	-.544	-.274	-.341	-.279
Rapid heart rate	Bo	.934	-.136	**.881**	-.170	.156	.293
Spoke more slowly	Ex	.934	-.114	**-.873**	.008	.130	-.377
Rapid breathing	Bo	.900	-.082	**.869**	-.035	.171	.328
Slowed heart rate	Bo	.955	-.170	**-.855**	-.004	-.190	-.400
Slowed breathing	Bo	.930	-.189	**-.854**	-.092	-.120	-.377
Wanted to do nothing	Be	.871	-.077	**-.817**	.340	.105	-.269
Tense muscles	Bo	.839	.222	**.795**	.015	.113	.381
Spoke more rapidly	Ex	.875	-.162	**.782**	-.219	-.232	.368
Tired	F	.904	.150	**-.780**	.432	-.138	-.259
Sweating	Bo	.812	.267	**.755**	.114	.258	.302
Spoke more loudly	Ex	.836	-.092	**.728**	-.162	-.409	.323
Closed the eyes	Ex	.855	-.180	**-.715**	-.101	.197	-.513
The event required an immediate response	Ev	.772	.513	**.612**	-.067	-.049	.357
Calm	F	.913	-.469	**-.601**	-.247	-.333	-.401
Wanted to tackle the situation	Be	.852	-.176	.324	**-.727**	-.428	.077
The event was important for and relevant to the person's goals or needs	Ev	.861	-.510	.152	**-.705**	-.253	-.130
The event was caused by somebody else's behavior	Ev	.706	-.126	.314	**.671**	.116	.357

Feature	Comp.	Comm.	Valence	Arousal	Power Motivation	Power Potential	Novelty
Wanted someone else to take the initiative	Be	.818	.061	-.573	**.671**	.156	-.105
Lacked the motivation to pay attention to what was happening	Be	.868	.459	-.477	**.638**	.052	-.138
Strong	F	.913	-.453	.189	**-.628**	-.522	-.066
Weak	F	.863	.345	-.323	**.617**	.496	-.114
Wanted to overcome an obstacle	Be	.765	.209	.519	**-.545**	-.391	.049
Awake	F	.807	-.402	.448	**-.497**	-.433	.095
Spoke in a trembling voice	Ex	.911	.183	.194	.143	**.884**	.193
Had tears in the eyes	Ex	.846	-.316	.123	-.140	**.775**	-.332
Spoke in a firm voice	Ex	.880	-.218	.178	-.448	**-.764**	-.127
Had speech disturbances	Ex	.809	.131	.181	.204	**.720**	.445
The person had a dominant role in the situation involving the event	Ev	.855	-.246	.088	-.511	**-.686**	-.236
Feeling weak in the limbs	Bo	.892	.264	-.411	.435	**.681**	.010
The person had control over the consequences of the event	Ev	.918	-.340	-.136	-.476	**-.660**	-.350
The person was powerless in this situation involving the event	Ev	.864	.388	.048	.519	**.649**	.146
Becoming pale	Bo	.889	.492	-.090	.424	**.645**	.206
The person had power over the consequences of the event	Ev	.857	-.328	-.063	-.512	**-.617**	-.321
The event confirmed the expectations of the person	Ev	.894	-.475	-.209	-.270	**-.548**	-.501
The event had consequences that were predictable	Ev	.733	-.265	-.334	.064	**-.526**	-.521
The event was uncontrollable	Ev	.823	.331	.291	.413	**.521**	.432
The event was caused by the persons own behavior	Ev	.753	-.199	-.101	-.429	**-.517**	-.502
Raised the eyebrows	Ex	.869	.221	.217	.055	-.108	**.871**

Feature	Comp.	Comm.	Valence	Arousal	Power Motivation	Power Potential	Novelty
Dropped their jaw	Ex	.824	-.071	.188	-.216	.132	**.848**
The event occurred suddenly	Ev	.833	-.173	.516	-.090	.071	**.724**
The event was unpredictable	Ev	.804	-.047	.494	.141	.138	**.721**
The event happened by chance	Ev	.755	-.394	.316	-.065	-.107	**.696**

Note. Comp. = Component according to the CPM. Comm. = Communality. F=Subjective Feeling, Bo=Bodily Reactions, Ex=Expression, Be=Behavior Tendencies, Ev=Event Evaluation. Boldface indicates the highest loading of each feature. The feature loadings indicate that the two dimensions valence and power are inverted: for *valence*, positive loadings represent negative valence; for both *power* dimensions, positive loadings stand for low power, and vice versa.

Supplementary Table 5

Cluster Centers for the 15 Clusters and Each GRID Feature

Feature	1	2	3	4	5	6	7	8	9	10	11	12	13	14	15
3 Good	0.87	-0.14	1.92	-2.68	2.21	2.12	1.12	2.81	0.55	-2.95	3.32	-2.90	3.17	-0.89	-3.21
4 Tired	-2.25	-0.29	-2.77	-1.30	-2.19	-1.59	-1.73	1.45	-2.28	-0.52	-0.67	-0.36	-1.57	3.29	-2.13
5 Restless	0.49	-0.29	0.46	1.99	-0.07	-0.75	0.33	-2.08	0.14	1.38	-1.70	1.43	-1.19	-0.29	1.90
6 Strong	0.29	-0.94	2.24	-2.12	1.75	1.11	1.27	0.69	0.24	-2.27	1.92	-1.30	2.04	-0.83	-0.35
7 Calm	-0.84	0.73	-1.29	-2.79	-0.57	0.80	-0.29	3.31	-1.42	-1.71	2.68	-1.76	1.14	1.21	-3.45
8 Bad	-1.04	0.35	-1.84	1.82	-2.33	-2.24	-1.45	-1.86	-1.15	2.33	-2.56	2.44	-2.84	1.23	1.66
9 Weak	-0.68	0.72	-2.34	0.95	-1.63	-1.38	-0.90	-0.45	-1.15	1.51	-1.24	0.39	-1.67	1.17	-0.36
10 Awake	1.42	-0.09	2.70	-0.31	2.33	1.33	2.31	0.26	1.36	-1.20	1.64	-0.08	2.07	-1.11	0.29
11 Feeling weak in the limbs	0.59	1.14	-2.21	1.25	-1.60	-0.22	-1.05	-0.15	-0.48	2.07	-1.64	0.62	-1.59	1.28	-1.21
12 Becoming pale	0.31	0.16	-1.93	1.58	-1.94	-1.26	-0.99	-1.84	0.51	1.86	-1.86	1.41	-2.01	-0.32	-0.12
13 Stomach disturbance	-0.67	0.09	-2.02	1.66	-2.13	-1.90	-1.29	-2.21	-0.49	2.40	-2.67	2.03	-2.52	-0.99	2.02
14 Slowed heart rate	-1.43	0.56	-2.34	-2.38	-1.69	-0.20	-1.48	3.28	-2.13	-0.76	1.48	-1.34	-0.61	2.84	-3.16
15 Rapid heart rate	1.89	0.26	2.50	1.92	2.01	1.08	1.26	-1.69	1.66	0.42	-0.19	0.74	1.72	-1.83	2.42
16 Tense muscles	0.76	-0.89	1.83	1.58	0.97	0.62	0.61	-1.86	1.27	0.45	-1.20	1.15	0.43	-1.60	2.27
17 Slowed breathing	-0.84	0.67	-2.28	-1.77	-1.22	0.40	-0.29	3.63	-1.92	-0.62	1.62	-1.13	-0.21	2.37	-2.40
18 Rapid breathing	1.42	-0.08	2.15	1.86	1.66	0.56	0.32	-1.75	1.47	0.05	-0.50	0.74	1.16	-1.71	2.45
19 Feeling warm	1.14	0.39	1.47	-1.59	1.74	1.98	0.53	2.80	0.24	-2.14	2.64	-2.03	2.70	-0.72	-0.95
20 Sweating	0.54	-1.04	0.88	1.31	0.31	-0.54	0.46	-1.89	0.57	0.76	-1.52	0.23	-0.23	-1.73	2.09
21 Feeling cold	-0.95	-0.05	-2.06	0.62	-2.04	-1.37	-1.44	-1.30	-0.97	1.76	-1.54	1.30	-2.25	1.06	-0.50
22 Smiled	0.65	-0.10	1.84	-2.90	2.67	2.46	0.42	2.50	0.32	-2.80	3.42	-3.26	3.56	-1.21	-3.12
23 Dropped their jaw	0.77	-1.70	0.32	0.68	0.55	0.66	-0.08	-1.69	3.17	-2.16	-1.52	0.24	-0.39	-1.76	0.11
24 Raised the eyebrows	-0.13	-1.61	0.39	0.92	0.26	-0.25	0.73	-1.60	2.62	-1.68	-1.27	0.62	-0.83	-0.74	0.23
25 Frowned	-1.38	-1.06	-0.99	1.30	-1.78	-1.16	1.00	-1.47	1.04	0.14	-2.00	2.03	-2.04	-0.06	1.06

Feature	1	2	3	4	5	6	7	8	9	10	11	12	13	14	15
26 Closed the eyes	0.64	2.28	-1.77	-0.82	-0.63	0.91	0.20	3.51	-2.36	0.52	1.64	0.17	0.72	2.36	-1.51
27 Had tears in the eyes	1.96	2.31	-0.69	0.03	0.01	0.47	-1.38	-0.96	-0.87	2.04	-0.16	-1.04	0.63	-1.61	0.48
28 Spoke more loudly	-0.20	-1.59	1.75	0.10	1.68	0.17	0.17	-1.77	1.36	-1.51	-0.19	0.04	1.11	-1.43	2.37
29 Spoke in a trembling voice	1.67	1.36	-0.72	1.94	-0.63	0.26	-0.67	-1.53	0.60	1.86	-1.55	0.08	-0.97	-1.50	1.44
30 Spoke in a firm voice	-1.20	-0.97	1.62	-1.82	1.37	0.66	0.97	0.40	-0.17	-2.12	1.83	-0.31	1.29	-0.38	0.10
31 Had speech disturbances	1.17	0.39	-0.59	1.47	-0.19	0.52	-0.06	-0.95	1.54	1.18	-1.21	0.03	-0.96	-0.84	1.68
32 Spoke more rapidly	0.33	-1.14	2.18	0.81	1.90	0.45	0.75	-1.77	1.60	-1.41	-0.67	-0.46	1.28	-1.68	2.26
33 Spoke more slowly	0.24	1.55	-1.95	-0.87	-1.29	0.50	0.11	3.13	-1.10	0.97	1.39	-0.53	-0.05	2.87	-2.25
34 Wanted the ongoing situation to last or be repeated	1.43	0.58	2.20	-2.74	3.11	2.62	1.03	2.64	0.76	-3.10	3.65	-3.47	3.76	-1.78	-2.64
35 Wanted to stop what he/she was doing	-0.91	-0.06	-1.31	1.09	-1.63	-1.56	-0.48	-1.02	-0.50	1.73	-1.30	1.76	-1.75	1.88	1.57
36 Wanted to undo what was happening	-1.22	0.58	-1.33	1.73	-2.35	-2.34	-0.83	-1.55	-0.63	2.32	-2.01	2.27	-2.60	0.63	2.02
37 Wanted to comply with someone else's wishes	-0.46	0.38	-1.01	-1.13	-0.14	-0.66	-0.52	-0.02	-0.77	-0.90	0.07	-1.06	0.08	0.36	-1.61
38 Wanted someone else to take the initiative	-0.36	0.41	-1.83	0.53	-1.04	-0.94	-1.16	0.95	-0.80	0.59	-0.52	0.20	-1.04	2.42	-1.21
39 Wanted to do nothing	-0.56	0.45	-2.72	-0.98	-1.67	-0.36	-1.77	2.18	-1.54	0.82	0.13	-0.24	-0.62	2.77	-1.95
40 Lacked the motivation to pay attention to what was happening	-1.51	-0.38	-2.48	0.23	-2.21	-1.76	-1.72	-0.01	-1.86	0.91	-1.41	1.01	-2.07	4.00	-0.60
41 Wanted to disappear or hide from others	-0.59	0.39	-2.11	1.61	-2.35	-1.93	-1.03	-1.54	-1.14	2.51	-1.97	1.96	-2.65	0.01	-0.35
42 Wanted to do damage, hit, or say something that hurts	-1.52	-0.99	-0.66	0.93	-2.08	-2.28	-1.37	-2.46	-0.56	0.67	-2.44	1.52	-2.63	-0.78	2.48

Feature	1	2	3	4	5	6	7	8	9	10	11	12	13	14	15
43 Wanted to oppose someone or something	-0.73	-0.78	0.26	0.58	-0.62	-1.01	0.08	-1.93	-0.13	0.86	-1.41	1.46	-1.51	-0.44	2.72
44 Wanted to tackle the situation	0.18	-0.35	1.93	-0.62	1.10	0.99	1.90	-0.33	0.50	-1.44	0.64	-0.78	1.28	-1.35	-0.18
45 Wanted to overcome an obstacle	-0.46	-0.47	1.44	-0.45	0.63	-0.08	1.41	-1.21	-0.10	-0.65	-0.09	-0.44	0.07	-0.79	0.60
46 Wanted to run away in any direction	-0.52	-0.04	-1.29	1.99	-1.98	-2.06	-1.04	-2.35	-0.51	2.11	-2.31	1.75	-2.42	-0.97	1.36
47 Wanted to sing and dance	0.08	-0.21	1.96	-3.09	2.64	1.71	-0.54	0.65	-0.19	-3.38	1.73	-3.31	2.87	-1.84	-2.79
48 The event occurred suddenly	1.00	0.04	1.40	1.46	1.37	1.17	1.20	-0.31	2.74	0.17	-0.01	0.49	0.80	-0.55	1.16
49 The event was unpredictable	0.77	0.50	1.35	1.49	1.29	0.54	0.77	-0.37	2.24	0.47	0.17	0.85	0.39	-0.38	1.10
50 The event confirmed the expectations of the person	-0.90	-0.71	-0.13	-2.15	0.32	0.35	-0.65	1.49	-2.12	-1.21	1.70	-1.71	1.28	0.65	-1.77
51 The event was pleasant for the person	0.93	0.02	1.96	-2.82	2.77	2.07	0.81	3.05	0.30	-3.09	3.54	-3.31	3.54	-0.85	-3.10
52 The event was important for and relevant to the person's goals or needs	0.98	0.63	2.01	-0.21	1.59	1.38	1.67	1.37	0.34	-0.99	1.78	-0.86	2.03	-1.29	-0.93
53 The event was important for and relevant to the goals or needs of somebody else	0.57	-0.30	0.83	-0.41	0.66	0.38	0.27	0.19	0.07	-0.94	0.56	-0.71	0.96	-0.10	-0.55
54 The event happened by chance	0.70	0.19	1.11	0.82	1.17	0.52	0.58	0.14	2.09	0.05	0.22	0.31	0.78	-0.14	0.26
55 The event was caused by the person's own behavior	-0.76	-0.41	0.12	-1.15	-0.08	-0.37	0.23	0.78	-1.64	-0.49	0.55	-1.08	0.59	-0.20	-0.71

Feature	1	2	3	4	5	6	7	8	9	10	11	12	13	14	15
56 The event was caused by somebody else's behavior	0.88	1.04	0.97	1.21	1.05	0.70	0.30	0.18	1.66	0.92	0.45	1.31	0.75	0.86	1.44
57 The event had consequences that were predictable	-0.42	-0.35	-0.62	-1.03	0.04	-0.27	0.14	1.18	-1.93	-0.74	1.21	-0.31	0.49	1.34	-1.24
58 The event had negative, undesirable consequences for the person	-0.83	0.09	-1.08	1.86	-2.15	-1.63	-0.51	-1.92	-0.30	2.81	-2.38	2.03	-2.75	-0.49	2.57
59 The event required an immediate response	-0.37	-0.68	1.49	0.99	0.63	-0.35	1.01	-1.21	0.79	0.21	-1.23	0.53	-0.64	-0.96	1.09
60 The person had power over the consequences of the event	-0.53	-0.94	0.68	-2.11	0.56	0.29	0.80	1.14	-1.28	-1.40	1.24	-1.16	0.94	-0.34	-1.41
61 The person had control over the consequences of the event	-0.71	-1.04	0.47	-2.31	0.50	-0.20	0.53	1.19	-1.39	-1.76	1.62	-1.57	1.13	0.27	-1.50
62 The person could live with the consequences of the event	1.00	0.34	0.43	-1.15	1.56	2.02	0.74	2.31	0.05	-0.80	2.55	-0.81	1.67	0.74	-1.76
63 The event was inconsistent with the person's own standards and ideals	-0.75	-0.08	-0.58	1.70	-1.57	-1.24	-0.59	-1.66	0.12	2.33	-1.85	2.36	-2.33	0.09	2.57
64 The event involved the violation of socially accepted norms	-0.48	-0.27	-0.05	1.20	-1.03	-1.15	-0.46	-1.63	1.15	2.32	-1.51	2.17	-1.92	-0.76	2.23
65 The person was powerless in this situation involving the event	0.59	0.96	-0.74	2.04	-0.69	0.22	-0.41	-0.79	0.53	2.46	-1.30	0.86	-1.33	0.47	1.76
66 The person had a dominant role in the situation involving the event	-1.04	-1.04	0.67	-1.95	0.60	-0.15	0.43	0.10	-0.72	-1.27	1.19	-1.31	0.74	-0.47	-0.50

Feature	1	2	3	4	5	6	7	8	9	10	11	12	13	14	15
67 There was no urgency in the situation involving the event	0.61	0.56	-0.26	-1.47	0.37	1.41	-0.33	3.04	-0.69	-1.36	2.05	-0.98	1.57	1.70	-1.64
68 The event was uncontrollable	0.36	0.98	0.45	1.84	0.14	-0.22	0.07	-1.19	1.71	1.40	-1.06	1.07	-0.57	-0.19	1.41

Note. Cluster names: 1 = moved/in awe, 2 = longing/melancholic, 3 = invigorated/interested, 4 = confused/worried, 5 = merry/attracted, 6 = fascinated/enchanted, 7 = intellectually stimulated, 8 = relaxed, 9 = surprised, 10 = sad, 11 = pleased/feeling harmony, 12 = displeased/repelled, 13 = delighted/feeling beauty, 14 = bored, 15 = angry.

Supplementary Table 6

Mean Jaccard Similarity Values

Cluster	Mean Jaccard similarity value
Moved / Awe	0.85
Longing / Melancholic	0.75
Invigorated / Interested	0.71
Confused / Worried	0.71
Merry / Attracted	0.71
Fascinated / Enchanted	0.74
Intellectually Stimulated	0.93
Relaxed	0.83
Surprised	0.90
Sad	0.72
Pleased / Harmony	0.68
Displeased / Repelled	0.89
Delighted / Feeling beauty	0.87
Bored	0.95
Angry	0.86

Supplementary Figure 1

Exemplary Illustration of the Aesthetic Emotion GRID task for one feature of one category.

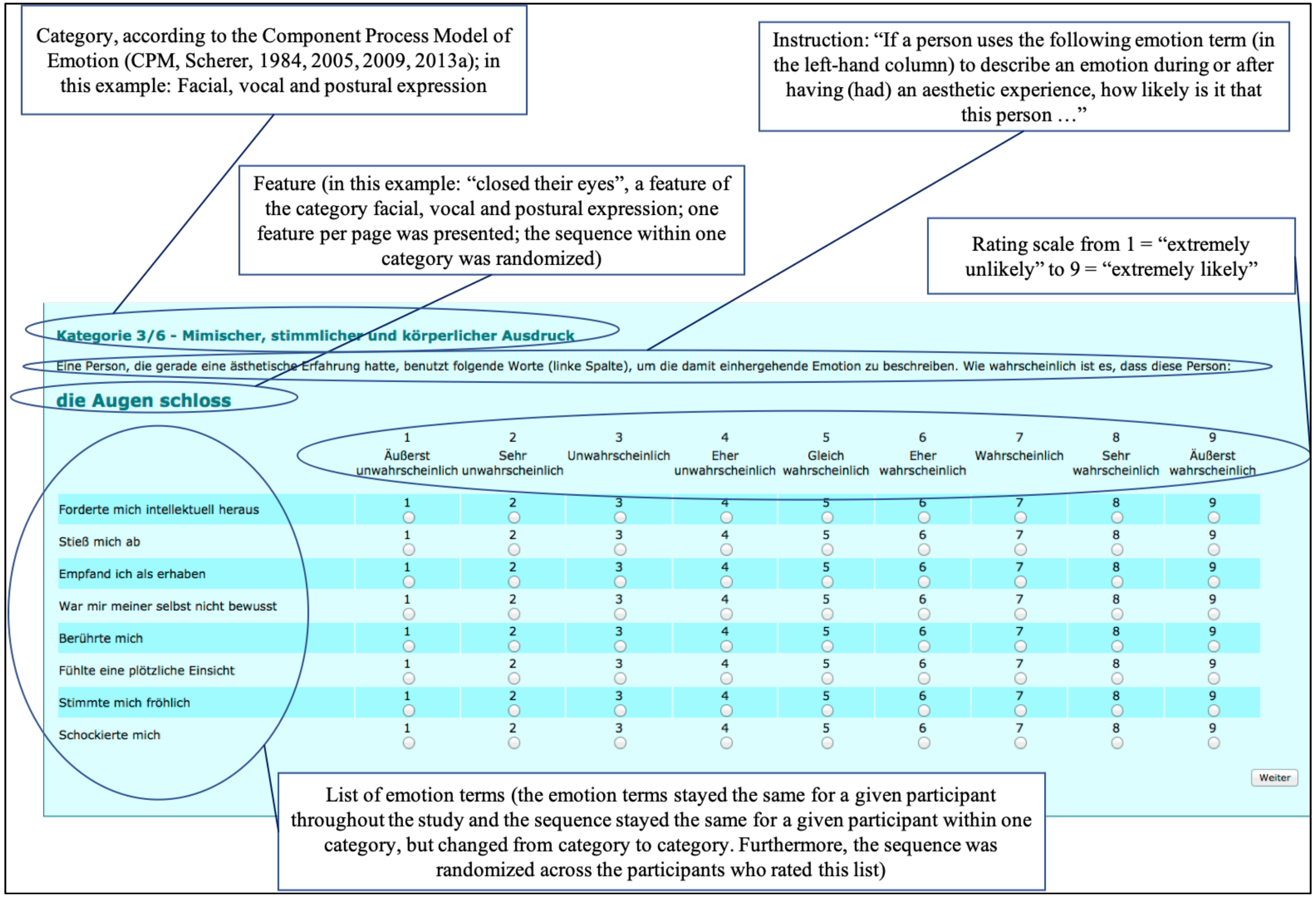

Supplementary Figure 2
Screeplot of the PCA on the Aesthetic Emotion GRID

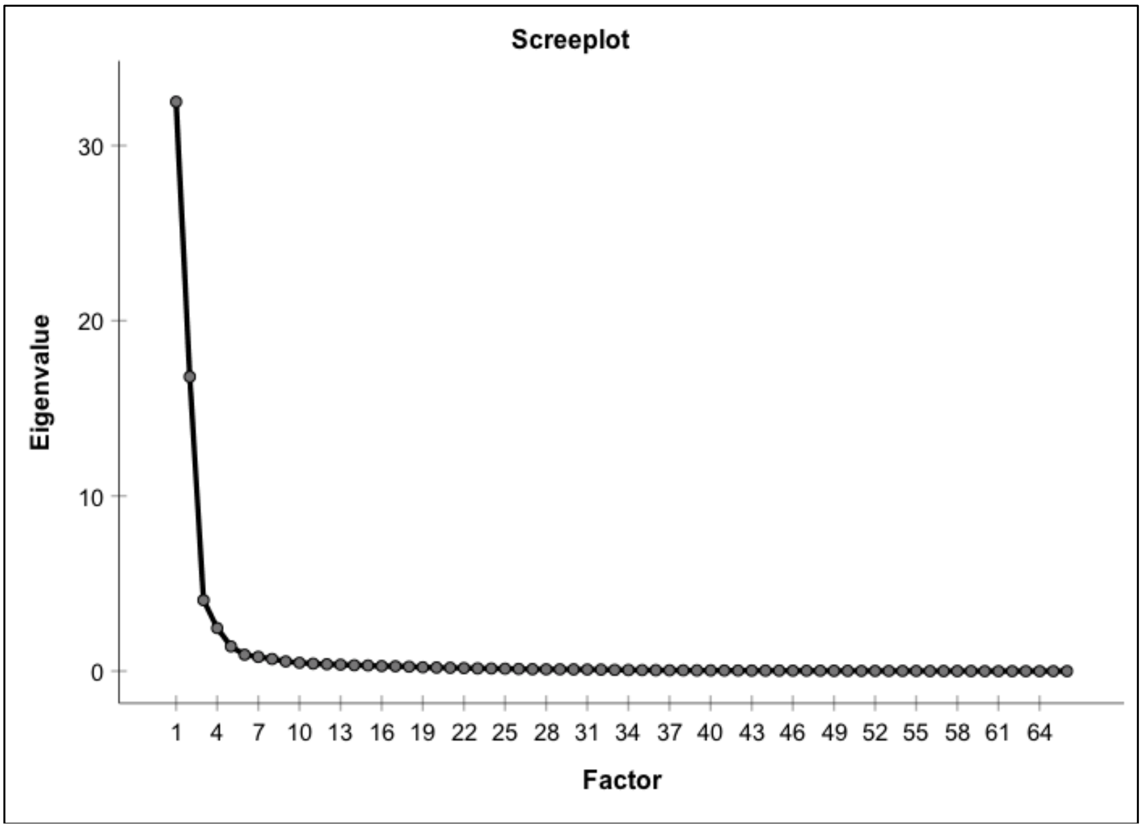

Supplementary Figure 3

Feature Scores of the 75 Emotion Terms Represented by the Four-dimensional Structure for Valence x Arousal (Panel A), Power x Arousal (B), and Valence x Novelty (C)

Panel A

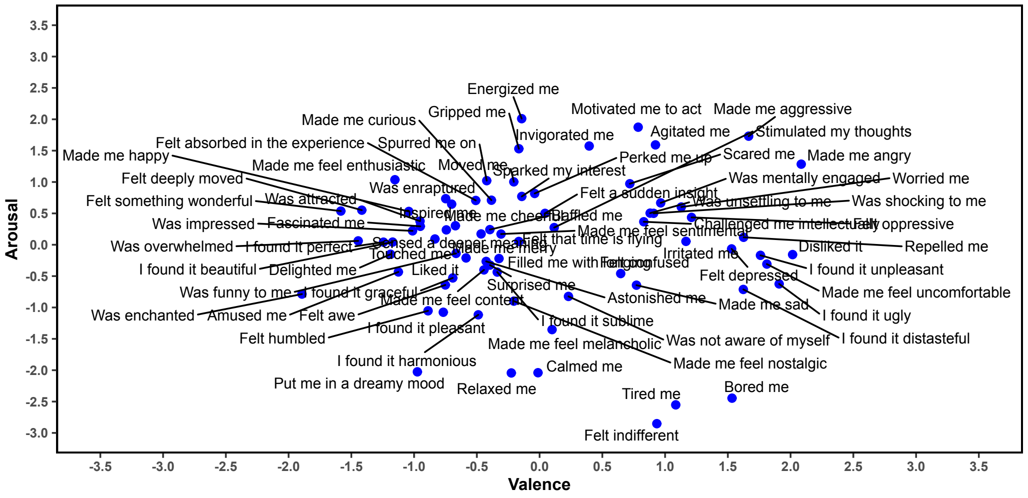

Panel B

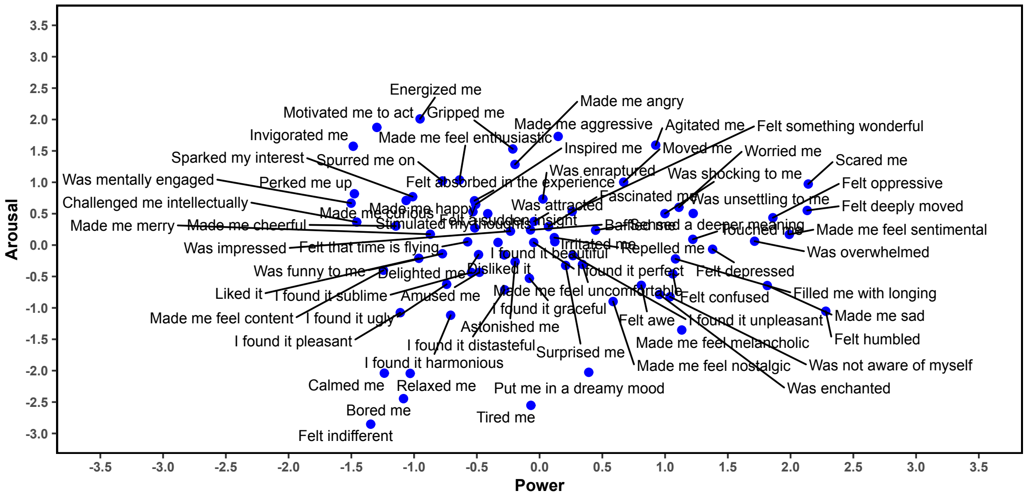

Panel C

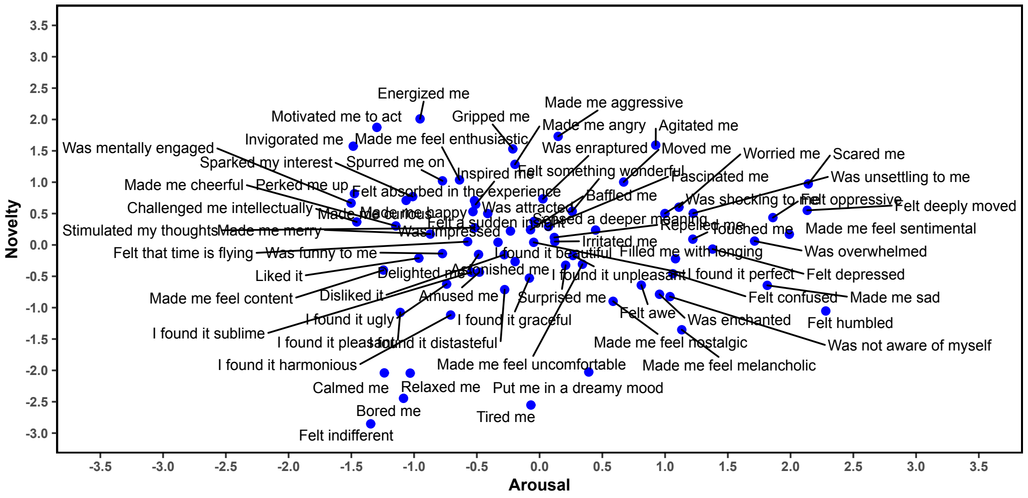

Supplementary Figure 4

The Gap Statistic for up to 50 Clusters Based on 100 Bootstrap Simulations per Number of Clusters

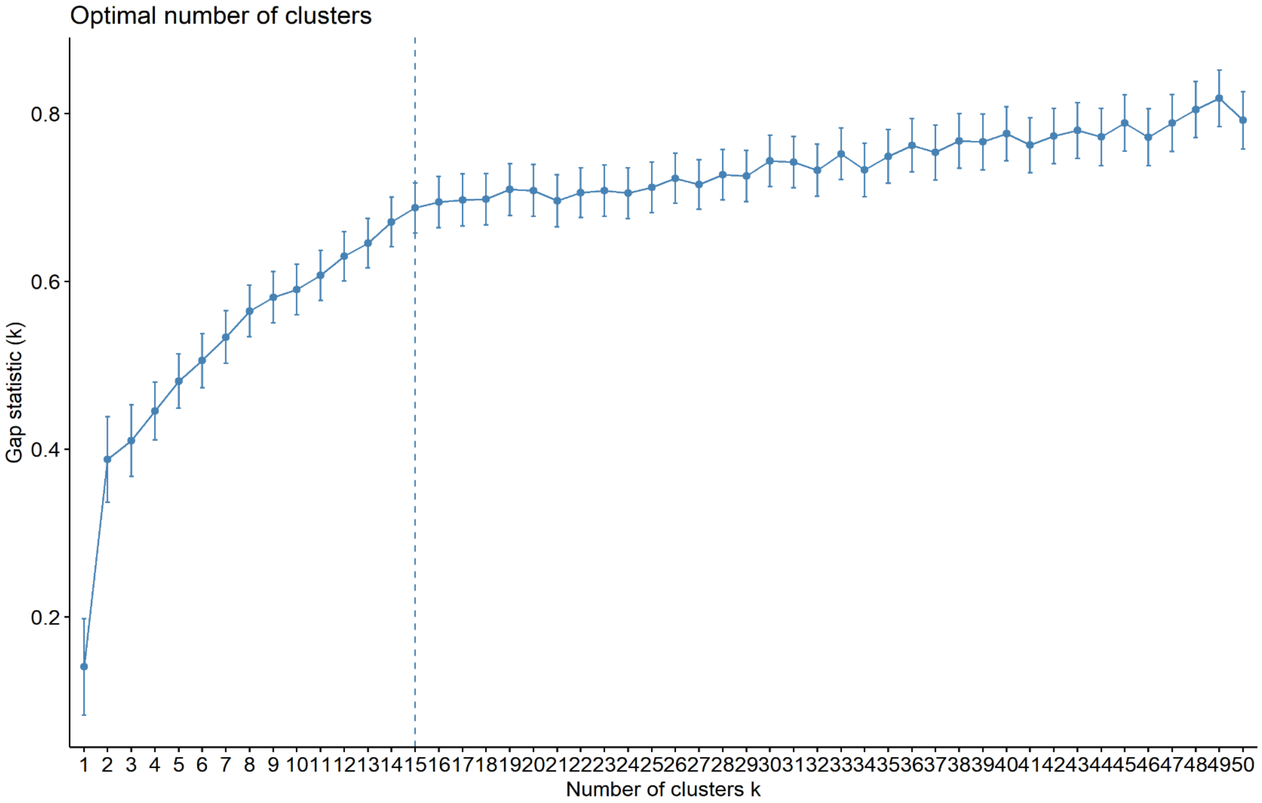

Note. The error bars represent the standard deviation of the simulation error. The optimal number of clusters based on the maxSE criterion as implemented in the R-package ‘factoextra’ is marked by a vertical, dotted line.

Cluster Analyses: Results of the Top and Bottom Five Features Characteristic for Each Cluster

Using exploratory *k*-means cluster analysis, we derived 15 clusters from the semantic feature profiles of 75 emotion terms as determined specifically for the context of aesthetics. The cluster center scores for each GRID feature are reported in Table S3. To determine the features that are particularly characteristic for each cluster, we identified the five GRID features with the highest scores and the five GRID features with the lowest scores for each cluster. These are illustrated in Figure 3 and 4. For the sake of a better overview of the results, the clusters in Figure 3 and 4 are allocated according to the approximate position in relation to the two dimensions in the cluster plot of Figure 2.

The features of the clusters 15 “angry”, 4 “confused/worried”, 12 “displeased/repelled”, and 10 “sad” (on the very left column of panels in Fig. 3)—support the interpretation of the low end of dimension 1 as negative valence in all five emotion components. Features representing low positive or high negative feelings, facial expressions, and event evaluations as well as a lack of appetitive or high avoidant behavior tendencies are characteristic of all four clusters. Specifically, the features “smiled” and “the event was pleasant for the person” were among the five GRID features with the lowest scores. Moreover, the cluster center scores for feeling “good” were among the lowest five scores in clusters 15, 12, and 10 and the sixth lowest in cluster 4 (see Table S3). The behavior tendency “wanted the ongoing situation to last or be repeated” was rated very low for clusters 4, 12, and 10 (it was ranked 7th among the low-scoring features for cluster 15). As a common feature for clusters 15, 12, and 10, the evaluation of the event as “inconsistent with the person’s own standards and ideals” was rated as high.

In contrast, analogue to Figure 2, the very right column of panels in Figure S5 presents three clusters that all include GRID features indicative of positive valence: clusters 5 “merry/attracted”, 13 “delighted/feeling beauty”, and 11 “pleased/feeling harmony.” For clusters 13 and 11, the subjective feeling “good,” the facial expression “smiled,” the behavior tendency “wanted the ongoing situation to last or be repeated,” and the evaluation “the event was pleasant for the person” represented four out of five GRID features with the highest scores. Smiling, seeking continued or repeated exposure, and evaluating the event as pleasant were also characteristic of cluster 5 (feeling good was ranked 6th; see Table S3).

The top clusters of Figure 2 and the top row of panels in Figure 3 and 4 represent the high end of dimension 2 (indicating high arousal) with four clusters. For clusters 15 “angry,” 9 “surprised,” 3 “invigorated/interested,” and 5 “merry/attracted,” subjective feelings of calmness (cluster 15) or tiredness (clusters 9, 3, and 5) as well as a “slowed heart rate” (clusters 15, 9, and 3) were considered uncharacteristic. Feeling “awake” was among the top-five high-scoring features in clusters 3 and 5.

The low end of dimension 2 is represented by two clusters included in the bottom row of clusters in Figure 2 and panels in Figure 3 and 4. Clusters 14 “bored” and 8 “relaxed” are clearly different in valence, as evidenced by a high score on feeling “tired” for cluster 14 and feeling “calm” for cluster 8. Nevertheless, both clusters share features indicative of low arousal: a “slowed heart rate” and “speaking more slowly.”

Lastly, the clusters 1 “moved/in awe,” 2 “longing/melancholic,” and 7 “intellectually stimulated” (middle part of Figures 2; middle right of Fig. 3, middle left of Fig. 4) seem to be insufficiently described by the two dimensions valence and arousal. As revealed by Figure 3 and 4, these clusters include a mixture of GRID features that might be cues to positive or negative valence. “Having tears in the eyes” and “speaking in a trembling voice” emerged as characteristic expressions in clusters 1 and 2, yet—unlike cluster 10 “sad”—the centers of both clusters do not show high scores for negative subjective feelings and avoidant behavior tendencies. On the contrary, the tendency to “want the ongoing situation to last or be repeated” was rated among the

top five GRID features in cluster 1. None of the most prominent features of cluster 2 indicate noticeable degrees of high or low arousal. Cluster 7 was characterized by feeling “awake” along with behavior tendencies to “tackle the situation” and to “overcome an obstacle,” while negative feelings or negative event evaluations were not characteristic of this cluster. To conclude, the analysis provides a structure of the aesthetic emotion terms into 15 clusters, based on similarities in the feature profiles of all five emotion components, that cover different nuances of aesthetic emotions.
